# ATR and CDK4/6 inhibition target the growth of methotrexate-resistant choriocarcinoma

**DOI:** 10.1038/s41388-022-02251-8

**Published:** 2022-03-18

**Authors:** Marina Georgiou, Panagiota Ntavelou, William Stokes, Rajat Roy, Geoffrey J. Maher, Tsvetana Stoilova, Josephine A.M.Y. Choo, Callum P. Rakhit, Miguel Martins, Paul Ajuh, Neil Horowitz, Ross S. Berkowitz, Kevin Elias, Michael J. Seckl, Olivier E. Pardo

**Affiliations:** 1 Division of Cancer, Department of Surgery and Cancer, Imperial College, London, UK; 2 MRC Toxicology Unit, University of Cambridge, Cambridge, UK; 3 Gemini Biosciences, Liverpool, UK; 4 Division of Gynecologic Oncology, Department of Obstetrics and Gynecology, Brigham and Women’s Hospital, Dana Farber Cancer Institute, Harvard Medical School, Boston, MA USA

**Keywords:** Cancer therapeutic resistance, Gynaecological cancer

## Abstract

Low-risk gestational trophoblastic neoplasia including choriocarcinoma is often effectively treated with Methotrexate (MTX) as a first line therapy. However, MTX resistance (MTX-R) occurs in at least ≈33% of cases. This can sometimes be salvaged with actinomycin-D but often requires more toxic combination chemotherapy. Moreover, additional therapy may be needed and, for high-risk patients, 5% still die from the multidrug-resistant disease. Consequently, new treatments that are less toxic and could reverse MTX-R are needed. Here, we compared the proteome/phosphoproteome of MTX-resistant and sensitive choriocarcinoma cells using quantitative mass-spectrometry to identify therapeutically actionable molecular changes associated with MTX-R. Bioinformatics analysis of the proteomic data identified cell cycle and DNA damage repair as major pathways associated with MTX-R. MTX-R choriocarcinoma cells undergo cell cycle delay in G1 phase that enables them to repair DNA damage more efficiently through non-homologous end joining in an ATR-dependent manner. Increased expression of cyclin-dependent kinase 4 (CDK4) and loss of p16^Ink4a^ in resistant cells suggested that CDK4 inhibition may be a strategy to treat MTX-R choriocarcinoma. Indeed, inhibition of CDK4/6 using genetic silencing or the clinically relevant inhibitor, Palbociclib, induced growth inhibition both in vitro and in an orthotopic in vivo mouse model. Finally, targeting the ATR pathway, genetically or pharmacologically, re-sensitised resistant cells to MTX in vitro and potently prevented the growth of MTX-R tumours in vivo. In short, we identified two novel therapeutic strategies to tackle MTX-R choriocarcinoma that could rapidly be translated into the clinic.

## Introduction

Gestational Trophoblastic Disease (GTD) comprises a group of pregnancy related disorders including the pre-malignant hydatidiform moles through to the malignant trophoblastic tumours [1, 2]. All GTDs arise from the trophoblastic elements of the placenta and they retain some of its properties such as the production of the beta human chorionic gonadotropin (βhCG) hormone. Currently, GTD affects approximately 1800 women per year in the UK, 24000 in Europe and more than 200,000 globally [3]. Malignant GTD incorporates four distinct pathological diagnoses: invasive moles, choriocarcinoma, epithelioid trophoblastic tumours (ETT) and placental-site trophoblastic tumours (PSTT) [3, 4]. Choriocarcinoma is the most aggressive of these, frequently forming distant metastasis to the lungs, vagina, liver, and/or brain. Choriocarcinoma may arise subsequent to any type of pregnancy including a molar pregnancy (~50%), a normal full-term pregnancy (22.5%), a spontaneous abortion (25%), or an ectopic pregnancy (2.5%) [5]. In Europe and North America, the incidence of choriocarcinoma is approximately 1 in 40,000 pregnancies, while in South East Asia, it is 9.2 in 40,000 pregnancies [6]. Due to the high vascularity of these tumours and their common high sensitivity to chemotherapy, surgery is often discouraged to avoid life-threatening haemorrhage [2]. Patients are stratified using the International Federation of Gynaecology and Obstetrics (FIGO) prognostic scoring and anatomical staging system to predict their risk of developing resistance to either (MTX) or actinomycin-D (ACT-D) [2]. The majority of the patients (~80%) fall into the low-risk group and are initially treated with MTX. However, this system misclassifies 30–40% of patients who are in fact MTX resistant (MTX-R) either from the outset (innate resistance) or during treatment (acquired resistance). These individuals then need further therapy either with ACT-D or much more toxic multi-agent chemotherapy in order to enter long-term remission [2], which is achieved in nearly 100% of cases. In contrast, the FIGO score high-risk patients all commence the etoposide, MTX and ACT-D alternating with cyclophosphamide and vincristine (EMA/CO) or similar toxic multi-agent therapies and if resistance occurs, then require further systemic treatments to achieve a 95% long-term remission rate. To prevent patients needing aggressive treatments like EMA/CO, less toxic new agents are needed to reverse MTX-R, which will likely arise from an improved understanding of the mechanisms involved.

Antifolates, such as MTX, impede nucleotide biosynthesis by directly binding and inhibiting enzymes such as thymidylate synthase (TS) or indirectly by blocking the folate cycle through inhibition of dihydrofolate reductase (DHFR) [7]. This leads to inhibition of DNA synthesis and results in subsequent DNA damage-induced cell death in cancer cells. Resistance to MTX has been observed in various cancers and several mechanisms of resistance have been reported over the past decades [7, 8]. Mechanisms include DHFR mutations that reduce its affinity for MTX or, more commonly in GTD, DHFR overexpression as a result of gene amplification or decreased miRNA targeting [7, 9–13]. However, DHFR expression levels alone failed as a consistent biomarker of MTX response in multiple cancers including GTD [14–16]. Similarly, numerous studies have failed to correlate the expression of several proteins involved in MTX response (e.g., FPGS, FPGH, RFC and TS) with the development of resistance [16–18].

Changes in DNA damage response (DDR) and cell cycle are crucial to cancer progression, including drug resistance [19, 20]. Accordingly, strategies to target these processes have been shown to improve therapeutic response [20, 21]. The ataxia telangiectasia and Rad3-related (ATR) signalling pathway has a central role in DDR and is often upregulated in cancers [22]. Targeting ATR itself, or its downstream mediators, CHK1 and WEE1, using small molecule inhibitors sensitised tumours to therapy in several clinical trials [23–28]. Similarly, inhibitors of the Cyclin-dependent Kinases (CDKs), such as the CDK4/6 inhibitor Palbociclib, are being used to target tumours with uncontrolled cell cycle progression [29, 30], for instance, following the loss of the CDK inhibitor p16^INK4a^ [31].

In the present study, we explored pathways associated with MTX resistance in choriocarcinoma cells using comparative proteomics and kinome-based RNA interference screening. Our data identified increased DNA repair and cell cycle deregulation in MTX-R choriocarcinoma cells, and we demonstrate that targeting these changes with clinically relevant small molecule inhibitors prevents the growth of resistant cells in vitro and in vivo. As these inhibitor drugs are already in clinical use, our findings could be rapidly translated to benefit patients.

## Results

### Methotrexate resistance in choriocarcinoma cells is associated with large scale proteomics changes

**Fig. 1:**
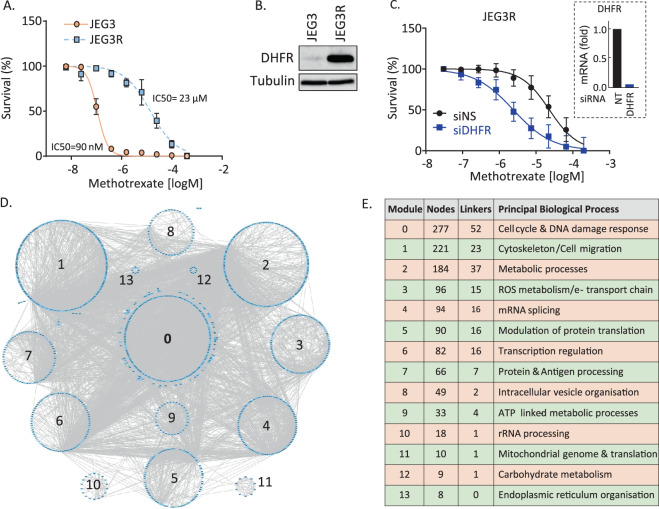
MTX resistance is associated with large scale proteomics changes in choriocarcinoma cells. **A** JEG3 and JEG3R cells were treated with increasing concentrations of MTX and cell survival determined by Crystal Violet staining. **B** DHFR was detected from lysates from exponentially growing JEG3 and JEG3R cells using Western blotting. Detection of β-Tubulin was used as a loading control. Representative blots of three biological replicates. **C** JEG3R cells silenced or not for DHFR expression were treated with increasing concentrations of MTX and cell survival determined by Crystal Violet staining. Insert: qPCR for *DHFR* demonstrates efficient target down regulation. **D**, **E** Comparative network generated by Reactome FI under Cytoscape from the SILAC-based total proteomics analysis of JEG3 and JEG3R cells. The network was further fragmented into 13 modules based on GO biological processes annotation (**D**). **E** GO biological processes associated with the corresponding network modules. The number of nodes and linkers for each GO processes category is shown. Nodes; proteins detected by MS/MS. Linkers; additional nodes introduced by Reactome FI to maximise network connectivity. **A**–**C** Data are normalised mean ± SEM.

**Fig. 2:**
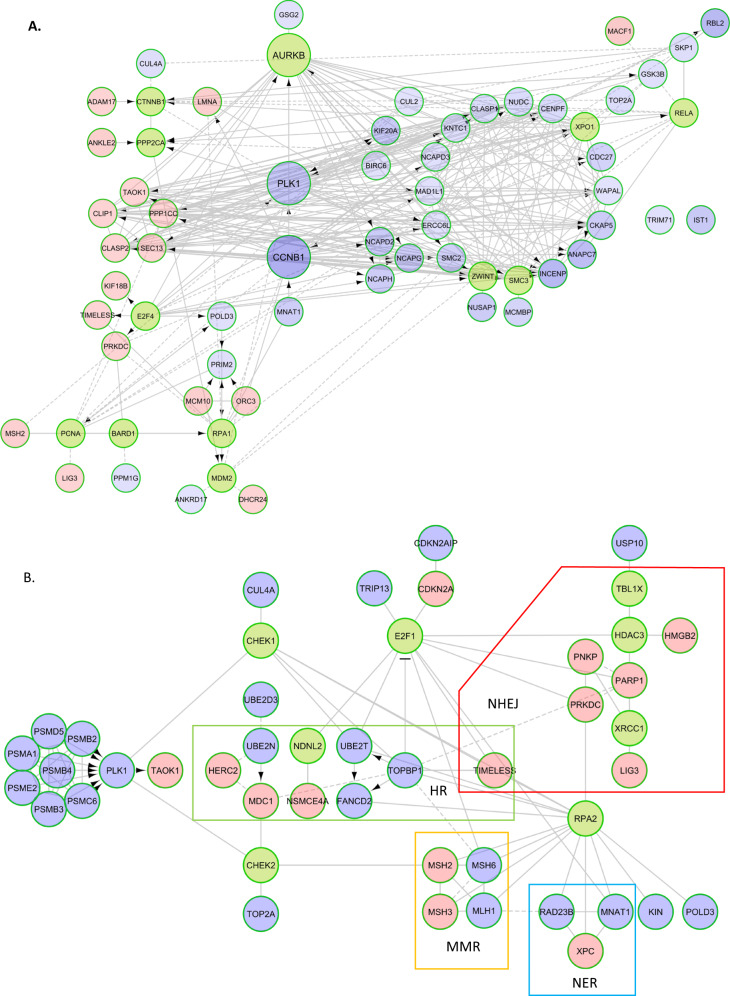
Functional interaction networks for cell cycle and DNA damage response. Cell cycle (**A**) and DDR (**B**) subnetworks. Blue nodes represent under-expressed and red overexpressed proteins in JEG3R compared to JEG3 cells. Green nodes are linkers. NHEJ Non-homologous end joining, HR Homologous recombination, MMR; mismatch repair, NER Nucleotide excision repair. Arrows; catalytic or transcriptional regulation, Plain lines; protein-protein interactions, Dashed lines; predicted protein-protein interactions.

The JEG3/JEG3R cell line pair is the only existing cell system modelling the MTX-sensitive/resistant status of choriocarcinoma. JEG3R cells were made resistant to MTX through long-term exposure of JEG3 cells to increasing concentrations of MTX over a 14-month period. Figure 1A illustrates that the IC_50_ for MTX is >250 times higher in JEG3R than JEG3 cells. This is associated with a large increase in the protein expression of DHFR, an intracellular target of MTX, in JEG3R as compared to JEG3 cells (Fig. 1B). Increased DHFR expression is a recognised molecular mechanism of acquired MTX resistance in various cancers [7]. However, siRNA-mediated silencing of DHFR only partially reverts the resistance of JEG3R cells to MTX (Fig. 1C), suggesting that additional molecular mechanisms are at play to induce this phenotype. To identify these, we performed quantitative SILAC-based mass spectrometric total and phospho-proteomics profiling of our cell line pair, which revealed large numbers of changes associated with MTX-resistance (Figs. 1D-E, 2 and Supplementary Fig. 1A-B, Supplementary Excel spreadsheet 1 and 2). To help reveal biological pathways and processes impacted by these changes, we performed functional network building using ReactomeFI [32] under Cytoscape (Fig. 1D) followed by Gene Ontology analysis (Fig. 1E and Supplementary Table 2). This analysis identified the biggest functional modules in our differential total proteomics network to be involved in DNA damage response (DDR) and cell cycle regulation (Module 0). Filtering Module 0 for hits directly involved in cell cycle regulation through literature mining enabled the building of subnetworks for the total and phosphoproteomics hits, respectively. Nodes coloured in blue showed decreased, and those in red increased, abundance (Fig. 2A) or phosphorylation (Supplementary Fig. 1A). Both subnetworks suggested decreased cell cycle progression in JEG3R as compared to JEG3 cells. Indeed, Fig. 2A shows decreased expression of two hyper-connected nodes central to cell cycle progression, CCNB1 and PLK1 [33, 34]. In contrast, levels of proteins CLIP1, a potent inhibitor of G1 cyclin-dependent kinases [33], and TAOK1 and CLASP2, which both delay cell cycle progression to enable DNA repair and mitotic fidelity [35, 36] were increased in JEG3R cells as compared to their MTX-sensitive counterparts. Similarly, the phospho-proteomics network shows decreased phosphorylation for hyper-connected nodes that promote cell cycle progression, such as CDK1 and CCNB1. In addition, we noted decreased phosphorylation of a large number of Aurora kinase B (AURKB) targets, suggesting that this kinase that is central to cell division [34] may have decreased activity in JEG3R cells. Similar filtering of Module 0 for hits directly involved in DNA damage response (DDR) enabled us to construct total and phospho-proteomics subnetworks (Fig. 2B and Supplementary Fig. 1B, respectively). JEG3R cells exhibit a general upregulation of hits involved with non-homologous end joining (NHEJ). In contrast, changes in the expression of proteins involved in homologous recombination (HR), mismatch repair (MMR) and nucleotide excision repair (NER) were more randomly modulated (Fig. 2B). This suggests that JEG3R could have more active NHEJ DNA repair than their MTX-sensitive counterparts. In addition, changes in the phosphorylation of proteins involved in HR suggest that this process may also be activated in MTX-resistant cells (Supplementary Fig. 1B). Indeed, phosphorylation of HMGB3, MTA1 and UHRF1 was increased in JEG3R cells and phosphorylation of these proteins on several sites has been shown to regulate their binding to DNA, repair abilities and/or subcellular localisation [37–41]. Taken together, our functional network analysis indicates a decreased cell cycle progression in JEG3R cells, which might enable these cells to undertake more efficient DNA repair as compared to JEG3 cells. As both decreased cell cycle progression and increased DDR would be expected to increase MTX-R, we decided to investigate this hypothesis further.

### Choriocarcinoma cells with MTX-R show decreased cell cycle progression

**Fig. 3:**
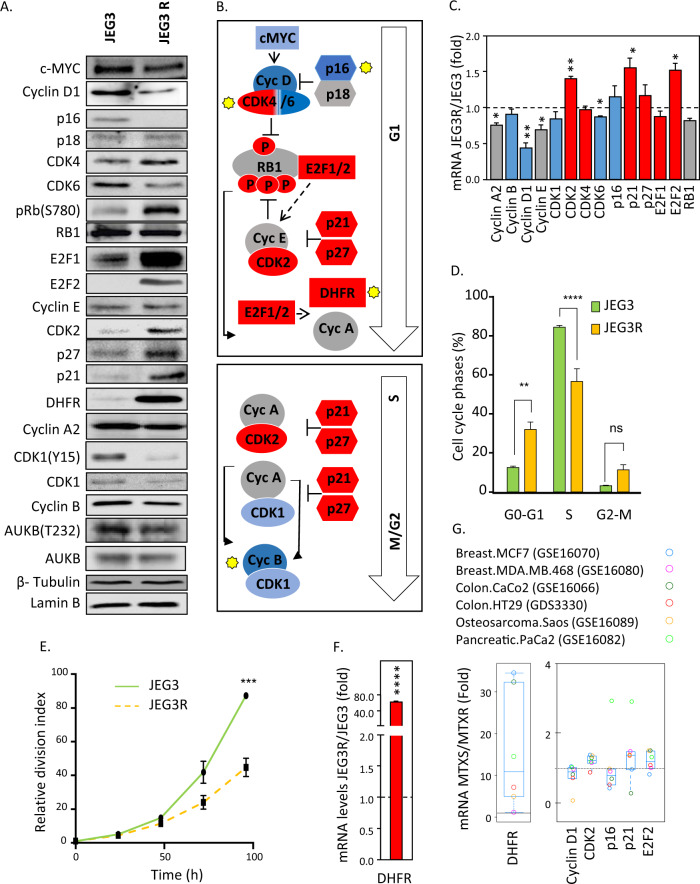
MTX-resistance is associated with delay in cell cycle progression. **A** Lysates from JEG3 and JEG3R were analysed by Western blotting for the indicated proteins with detection of Lamin B or Tubulin used as a loading control. Blots representative of 3 biological replicates. **B** Pathway diagram summarising changes in cell cycle proteins from (**A**) and total proteomics data. Box colours: Red; increased, Blue; decreased, Grey; unchanged. Yellow stars indicate Western blotting-validated MS data. **C** Expression levels for the indicated genes was determined using qPCR and fold changes in JEG3R vs JEG3 represented as mean ± SEM. Colours of bars represent associated changes at protein levels as seen in (**A**, **B**): Red; increased, Blue; decreased, Grey; unchanged. **D** Cell cycle profiles of JEG3 and JEG3R cells were analysed by flow cytometry following propidium iodide labelling. Bar graph represent percent of cells in each cell cycle phase as mean ± SEM of *n* = 3. **E** JEG3 and JEG3R cells were labelled using CFSE and tracked for 96 h with samples analysed every 24 h using flow cytometry. Geometric means of the CFSE fluorescence at each time point was normalised to the 0 h measurement and the inversed normalised fluorescence intensity values used to provide the relative division index. **F** Expression levels for DHFR was determined using qPCR and fold changes in JEG3R vs JEG3 represented as mean ± SEM. **G** GEO microarray datasets for various MTX sensitive/resistant cell line pairs were analysed for their expression of the indicated genes. Data are presented as fold change in resistant vs sensitive cells. Horizontal bar represents no change.

We next tested the hypothesis that cell cycle progression was impaired in JEG3R as compared to JEG3 cells by validating and extending our mass-spectrometry data. Western blotting was performed for a large number of proteins covering all phases of the cell cycle (Fig. 3A). Figure 3B summarises changes in their expression or phosphorylation in JEG3R, with yellow stars indicating agreement with mass spectrometric data. Our data highlight a pronounced decrease in the expression of cyclins and cyclin-dependent kinases (CDKs) that promote cell cycle progression such as Cyclin D1, CDK6, CDK1 and Cyclin B. In contrast, the levels of inhibitors of cyclin/CDK complexes, such as p21 and p27 were increased. Moreover, our phospho-proteomics network suggested that the activity of linker node AURKB may be decreased in JEG3R cells as the vast majority of its substrates detected in our experiment showed reduced phosphorylation (Supplementary Fig. 1A). Our Western blotting data confirmed this hypothesis as JEG3R cells showed reduced phosphorylation of T232 on AURKB, a site that regulates its activity (Fig. 3A). Taken together, these changes would be expected to result in delayed cell cycle progression. In keeping with this notion, our functional networks suggested that the activity of the transcription factor E2F1 might be impaired in JEG3R as compared to JEG3 cells (Fig. 2B). However, Western blotting revealed this protein to be massively overexpressed in JEG3R cells (Fig. 3A). This was not the result of increased mRNA transcription (Fig. 3C) nor of stabilisation of the protein (Supplementary Fig. 1C) and therefore suggests increased translation efficiency of existing mRNA as a possible mechanism. Contrary to the network data, these results would propose enhanced rather than suppressed cell cycle progression. Nevertheless, when we examined the activity of E2F1 using luciferase-based reporter assays, we found this to be decreased by ≈60% in JEG3R as compared to JEG3 cells (Supplementary Fig. 1D), despite hyperphosphorylation of RB on S780 that should release E2F1 to promote its activity. The decreased activity of E2F1 was not due to mutation in its coding sequence, as sequencing of the protein-coding regions of this gene revealed it to be wild-type (Supplementary Sequencing files). So, we next analysed the phosphorylation of S337 and S364, two sites known to regulate the activity of E2F1 [42, 43]. This revealed that the relative phosphorylation of E2F1 on S364 was decreased in JEG3R as compared to JEG3 cells, which may explain, at least in part, the decreased activity of this transcription factor in the resistant cell line (Supplementary Fig. 1E). Moreover, in support of the latter findings, cell cycle profiling of JEG3 and JEG3R revealed an accumulation of JEG3R cells in the G0/G1 phase of the cell cycle, with a corresponding decreased proportion of cells in S phase (Fig. 3D). This was associated with a reduced rate of cell division in JEG3R cells as assessed by CFSE-based pulse-chase assay (Fig. 3E and Supplementary Fig. 1F). In summary, proteomics changes associated with MTX-R in JEG3R cells result in cell cycle delay and decreased cell division.

Because the JEG3/JEG3R cell line pair is the only existing comparative model for MTX-R in choriocarcinoma, we interrogated publicly-available Gene Expression Omnibus (GEO) datasets for other cancer types to assess whether some of our observed changes were generic to this process. As the available GEO datasets comparing MTX-R and MTX-sensitive (MTX-S) samples reported mRNA levels for our genes of interest, we first studied how our proteomics changes correlated with those in mRNA in our cell line pair. The increase in DHFR protein observed in JEG3R cells (Fig. 1B) was associated with a dramatic increase in the levels for the corresponding mRNA (Fig. 3F). Amongst proteins showing increased expression in JEG3R (Fig. 3A), CDK2, p21 and E2F2 also had increased mRNA levels in JEG3R, while lower Cyclin D1 and CDK6 mRNA levels were associated with decreased expression of the corresponding proteins (Fig. 3C). Hence, we assessed whether these six genes showed similar expression changes upon acquisition of MTX resistance in cancer cell lines of various origin. Amongst these, five (DHFR, Cyclin D1, CDK2, p21 and E2F2) showed median expression changes consistent with those observed in JEG3R cells (Fig. 3G). In addition, p16, the most dramatically downregulated protein in JEG3R cells despite unaltered transcription of its gene (Fig. 3A, C), showed decreased mRNA expression in four out of six MTX-R cell lines (Fig. 3G). Hence, many molecular changes in cell cycle players associated with MTX-R in JEG3R appear conserved across additional cell systems.

### MTX-R choriocarcinoma cells display an ATR-dependent increase in NHEJ

**Fig. 4:**
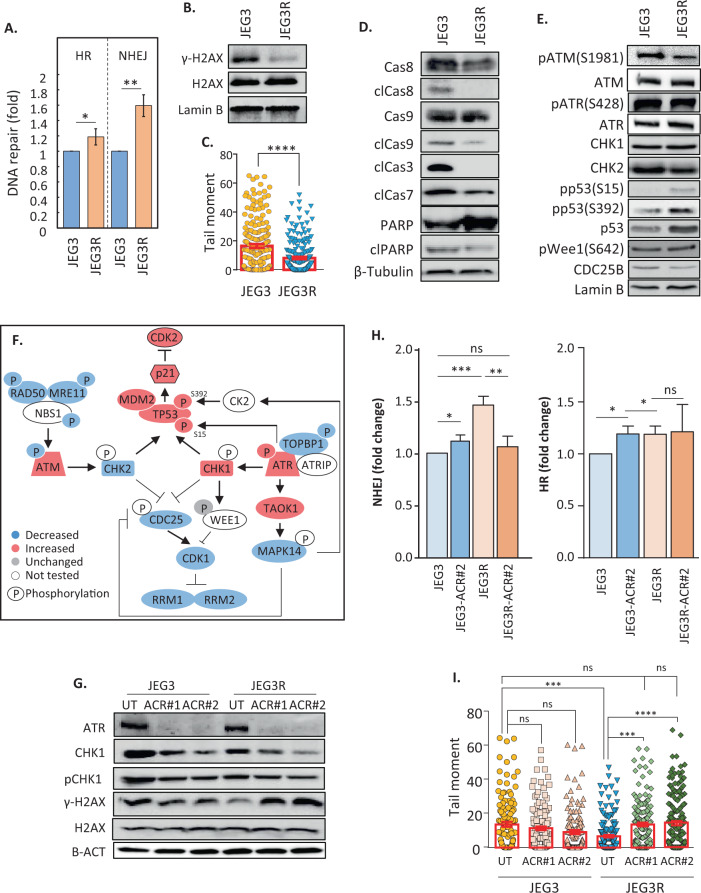
MTX resistance in JEG3 choriocarcinoma cells is associated with ATR-mediated changes in DNA damage response. **A** Reporter plasmid-based DNA damage repair assays reveals an increase in NHEJ over HR in JEG3R cells. **B** Western blotting of JEG3 and JEG3R cell lysates for the indicated proteins. Detection of Lamin B serves as loading control. Results representative of three biological replicates. **C**, **I** Comet assays performed on JEG3 and JEG3R cells (**D**) as well as their ATR CRISPR (ACR) or untargeted (UT) counterparts (**I**) with tail moment determined as a measure of background DNA damage. **D** *n* = 200 and (**I**) *n* = 100 comets per group were measured. **I** Two CRISPR clones per cell lines (#1 and #2) were tested. **D**, **E** Lysates from JEG3 and JEG3R cells were compared using Western blotting for background level of caspase activation (**D**) and proteins involved in the ATR and ATM pathways and their post-translational modifications (**E**). **D** The prefix ‘cl’ indicates cleaved versions of the target. Blots representative of three biological replicates. **F** The ATR rather than the ATM pathway appears activated in JEG3R cells. Diagram summarising changes in protein expression and post-translational modification obtained from Western blotting (**E**) and quantitative proteomics. **G** Western blotting for indicated proteins in untargeted (UT) and two separate ATR CRISPR knockout clones (#1 and #2) in JEG3 and JEG3R cells. Detection of β-Actin (B-ACT) was used as a loading control. **H** Effect of ATR knockout on DNA repair by HR and NHEJ was assessed using reporter plasmid-based DNA damage repair assays. Statistics: (**A** and **C**) Student *t*-test and (**H** and **I**) ANOVA. ****p < 0.001, ***p < 0.005, *p < 0.05, ns; *p* > 0.05.

Our functional network analysis suggested that JEG3R cells may have increased DNA repair activity, a phenomenon actively involved in MTX-R in colon cancer cells [44] (Fig. 2B). Therefore, we first decided to test this hypothesis by performing Western blotting for proteins involved more particularly in HR, MMR and NHEJ. These experiments showed that while there were decreases in the levels of MRE11 (involved in HR - Supplementary Fig. 2A), and MHL1 and MHS6 (involved in MMR - Supplementary Fig. 2B), two proteins involved in NHEJ, LIGIV and XRCC4, showed increased expression in JEG3R as compared to JEG3 cells (Supplementary Fig. 2C). To test whether JEG3R cells showed changes in DNA damage repair, we used a reporter plasmid-based assay to measure the ability of cells to repair DNA through these DDR pathways [45] (Supplementary Fig. 2D). This demonstrated that while a small increase in HR was noticeable between sensitive and resistant cells, there was a more pronounced increase in NHEJ activity in JEG3R cells (Fig. 4A and Supplementary Fig. 2E). This increased DNA repair activity was associated with lower baseline levels of DNA damage in JEG3R cells as assessed by decreased γ-H2AX levels (Fig. 4B) and a reduced tail moment from comet assays (Fig. 4C and Supplementary Fig. 2F). These changes were accompanied by inhibition of caspase 3, 7, 8 and 9 cleavage as well as that of the caspase 3 substrate PARP (Fig. 4D). P53 is a known mediator of apoptotic cell death downstream of DDR [46] and its activity is known to be regulated through a series of phosphorylation events [47]. As our phosphoproteomics data revealed increase in p53 Ser15 phosphorylation (Supplementary Fig. 1B and Supplementary Table 3), a site targeted by ATM or ATR kinases [47, 48], we investigated further possible changes in this pathway between JEG3 and JEG3R cells. We validated the increase in Ser15 phosphorylation of p53 in resistant cells, and further revealed increase in S392 phosphorylation of this protein (Fig. 4E). Both these sites serve different purposes. Ser15 phosphorylation disrupts binding of MDM2 to p53, leading to decreased proteosomal degradation of the latter protein [49]. In support for this, our results show accumulation of p53 in JEG3R cells that occurs through protein stabilisation (Fig. 4E and Supplementary Fig. 2G). In addition to this, our targeted-sequencing results revealed that p53 was not mutated on any common hotspots in JEG3R cells (Supplementary Table 3) and would therefore be expected to carry its wild-type functions. In agreement with this, one of the transcriptional targets of p53, p21 was also increased in JEG3R cells (Fig. 3A) suggesting that p53 accumulation plays a role in the cell cycle delay observed in these cells. In contrast, S392 phosphorylation promotes p53 localisation to the mitochondria and induction of transcriptional-independent apoptosis [50], which we did not functionally observe. Hence, our data suggest that despite p53 being stabilised and primed, this does not translate into apoptosis induction in MTX-R cells, but rather in cell cycle delay.

In addition, combined analysis of our proteomics (Supplementary Tables 2, 3) and Western blotting data (Figs. 3A, 4E) revealed that while the ATM pathway appeared downregulated in JEG3R as compared to JEG3 cells, the ATR pathway mediators and/or their phosphorylation were upregulated in the MTX-R cells (Fig. 4F). Consequently, this pathway might mediate the increased DNA repair observed in JEG3R cells. Indeed, CRISPR-mediated knockout of ATR in JEG3R cells cancelled out the difference in NHEJ but did not impact HR in resistant cells (Fig. 4G, H). In contrast, similar genetic ablation of ATR in JEG3 cells lead to a small increase in both HR and NHEJ (Fig. 4G, H). Consistent with the change in NHEJ in JEG3R cells, knockout of ATR increased the baseline DNA damage in these cells to levels indistinguishable from those found in JEG3 cells, as assessed by Western blot for γ-H2AX (Fig. 4G) and comet assay (Fig. 4I). In short, our data show that MTX-R is associated with increased ATR-mediated NHEJ in JEG3R cells.

### CDK4/6 inhibition with palbociclib inhibits the growth of JEG3R cells in vitro and in vivo

**Fig. 5:**
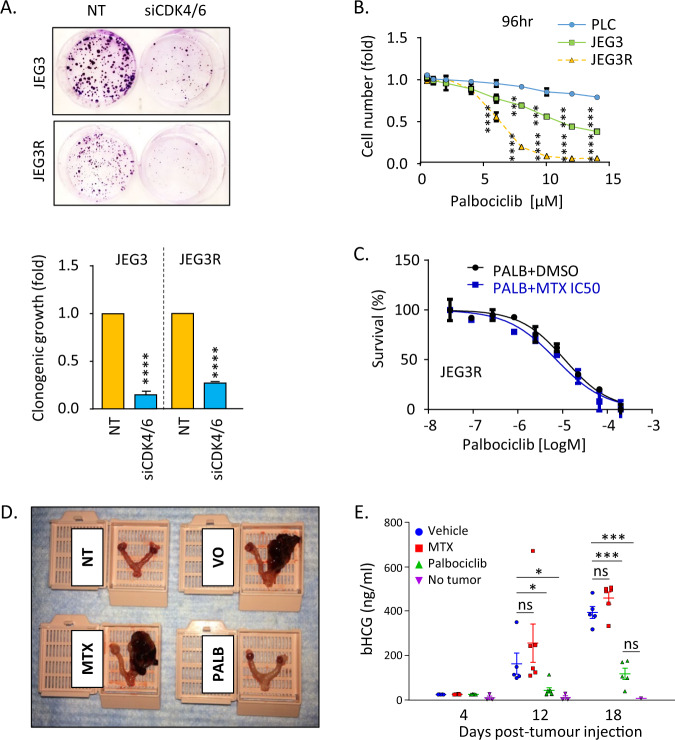
CDK4/6 inhibition inhibits the growth of JEG3R cells in vitro and in vivo. **A** JEG3 and JEG3R cells silenced (siCDK4/6) or not (NT) for CDK4 and six using siRNAs were subjected to clonogenic growth assay. Top panel: representative picture of clonogenic assay dishes stained with Crystal Violet. Bottom panel: colony numbers as a bar graph of mean ± SEM of three independent experiments performed in triplicates. **B** Normal placental (PLC), JEG3 and JEG3R cells were grown in the presence of increasing concentrations of Palbociclib and cell survival determined at 96 h using Crystal violet staining. Graph represents mean ± SEM of biological triplicates with *n* = 2 normalised to the corresponding untreated control. **C** JEG3R cells were treated with 23 µM MTX or DMSO (diluent control) together with increasing concentrations of Palbociclib. Cell survival was determined by Crystal Violet staining. Data are mean ± SEM of biological triplicates with *n* = 4 normalised to the corresponding Palbociclib untreated control. **D**, **E** JEG3R cells were injected at the uterine horn of *nude* mice. Mice received either Vehicle-only (VO) and Palbociclib (PALB-125mg/kg) or Methotrexate (MTX-1mg/kg). **D** Representative pictures from ex vivo uteri showing tumour burden. NT; animals not injected with tumour cells. **E** Blood collected on indicated days was analysed for β-hCG levels by ELISA. Data representative of experiments performed in duplicate. Statistics: Student *t*-test. ****p < 0.001, ***p < 0.005, **p < 0.01, *p < 0.05, ns non-significant.

Our results shown in Fig. 3A highlighted the loss of the cell cycle inhibitor p16 and accompanying increase in CDK4 expression in JEG3R as compared to JEG3 cells. As p16 is an inhibitor of CDK4 and 6, we assessed the impact of siRNA-mediated silencing of these two kinases on the clonogenic growth of our choriocarcinoma cell lines. We found that downregulating of these kinases impaired the growth of both JEG3 and JEG3R cells over a period of 2 weeks (Fig. 5A). Since both p16 down regulation and CDK4/6 overexpression are biomarkers of response to the CDK4/6 small molecule inhibitor, Palbociclib [51], we tested whether our MTX-resistant cells were more sensitive to this compound. Indeed, JEG3R cells appeared more sensitive to Palbociclib than their MTX-sensitive counterparts (Fig. 5B). Moreover, Palbociclib showed very low toxicity on the normal placental cell line PLC, suggesting a degree of cancer specificity to this compound that may provide a welcome therapeutic window in vivo. As decreased p16 expression may be commonly associated with MTX resistance (Fig. 3A, G), we tested whether Palbociclib could sensitise JEG3R cells to MTX. However, our results did not support this notion (Fig. 5C), suggesting that p16 down regulation and deregulation of CDK4/6 activity, while accompanying MTX resistance, are not instrumental to this phenotype. Hence, Palbociclib may be a novel monotherapy for the treatment of MTX-resistant choriocarcinoma. We tested this in an in vivo JEG3R orthotopic mouse model where single agent Palbociclib was compared to MTX in its ability to control tumour development. A total of 2 weeks following treatment initiation, ex vivo examination showed that Palbociclib efficiently prevented the macroscopic growth of tumours in the uterus while MTX was unable to control the disease (Fig. 5D). This was supported by measurements of circulating βhCG levels, a clinical marker of GTD progression. Indeed, blood levels for this hormone increased steadily during the timeframe of the experiment in the vehicle treated condition with no significant difference achieved following MTX treatment (Fig. 5E). In contrast, Palbociclib treatment significantly blunted that increase, with no difference observed at day 12 in βhCG levels as compared to mice devoid of tumours, and only a non-significant trend towards increased levels at day 18. The ability of Palbociclib to prevent disease progression was further confirmed by measurement of uterine weight at day 18 (Supplementary Fig. 3). Taken together, our data suggest that CDK4/6 inhibition is able to control the growth of MTX-resistant choriocarcinoma cells and that Palbociclib, used as monotherapy, could represent a novel therapeutic strategy for patients with MTX-R.

### ATR pathway inhibition sensitises JEG3R cells to MTX

**Fig. 6:**
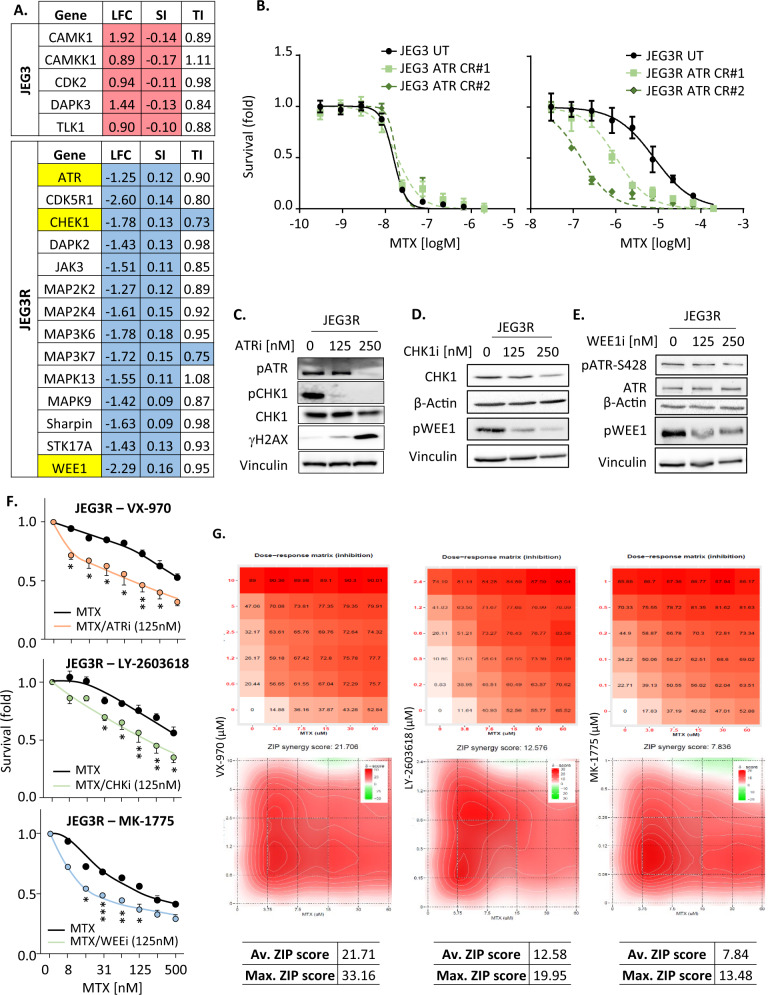
Inhibiting the ATR pathway re-sensitises JEG3R cells to MTX. **A** JEG3 and JEG3R cells were subjected to a kinome-wide siRNA screen in the presence and absence of MTX used at the IC_50_ of the corresponding cell line. Changes in cell survival were monitored by Crystal Violet staining. Table: LFC; Log2 fold changes in cell number in response to MTX following silencing of the indicated target as compared to non-targeted siRNA-transfected cells, SI sensitivity index to MTX calculated as in [87], TI toxicity index as fold changes in cell number following silencing of the indicated target in the absence of MTX. **B** JEG3 and JEG3R cells and their corresponding ATR CRISPR clones (#1 and 2) were treated with a dose range of MTX for 72 h before being subjected to Crystal Violet staining. **C**–**F** JEG3 or JEG3R cells were treated with the indicated concentrations of ATR (VX-970), CHK1 (LY-2603618) or WEE1 (MK-1775) inhibitors (shown as ATRi, CHKi and WEEi, respectively). **C**–**E** Cell lysates were subjected to Western blotting for the indicated targets. Detection of Vinculin was used as a loading control. Results representative of three biological replicates. **F** Cells were additionally treated with/without IC_50_ of MTX. Cell survival changes were revealed using Crystal Violet staining. Data are fold changes of mean ± SEM of representative experiments from biological triplicates with *n* = 4. **G** ZIP analysis for synergistic interaction between the indicated inhibitors and MTX. Upper panels; Dose-Response matrices. Lower panel, ZIP score contour line plots. Tables show the average and maximum synergy scores. Statistics: (**F**) Student *t*-test. ***p < 0.005, **p < 0.01, *p < 0.05, ns not significant.

Considering that ATR inhibition reverses increased NHEJ-mediated DNA repair in JEG3R cells, resulting in increased background DDR, we hypothesised that targeting ATR would sensitise resistant cells to MTX. In support of this idea, a kinome siRNA screen performed in our lab to identify modulators of response to MTX in the JEG3/JEG3R cell line pair revealed that silencing of 3 enzymes from the ATR pathway sensitised JEG3R cells to MTX (Fig. 6A and Supplementary Excel spreadsheet 3). The role of ATR in modulating responsiveness to MTX was validated using our ATR CRISPR cell lines. While ATR knockout did not increase the growth-inhibitory effects of MTX in JEG3 cells, both JEG3R ATR CRISPR clones showed sensitisation to MTX as compared to their untargeted CRISPR control (Fig. 6B). Similar results were obtained following the CRISPR-mediated knockout of CHK1, which re-sensitised JEG3R cells to MTX to levels equivalent to JEG3 cells (Supplementary Fig. 4A-B). Sensitisation to MTX was also achieved in JEG3R cells through inhibition of ATR, CHEK1 or WEE1 kinase activity using clinically-tested small molecule compounds (Fig. 6C-F). Those were used at 250 nM, a concentration that efficiently targeted the pathway as demonstrated by inhibition of ATR autophosphorylation by VX-970 (Fig. 6C) and WEE1 phosphorylation by LY-2603618 and MK-1775 (as this compound prevents autophosphorylation of this enzyme [52]). Also, consistent with results previously obtained following ATR knockout (Fig. 4H), ATR inhibition with VX-970 promoted background DNA damage in JEG3R as shown by an increase in γH2AX (Fig. 6C). The growth inhibition obtained through combining the ATR pathway inhibitors with MTX in JEG3R was synergistic as demonstrated using the Zero interaction potency (ZIP) model [53] (Fig. 6G).

**Fig. 7:**
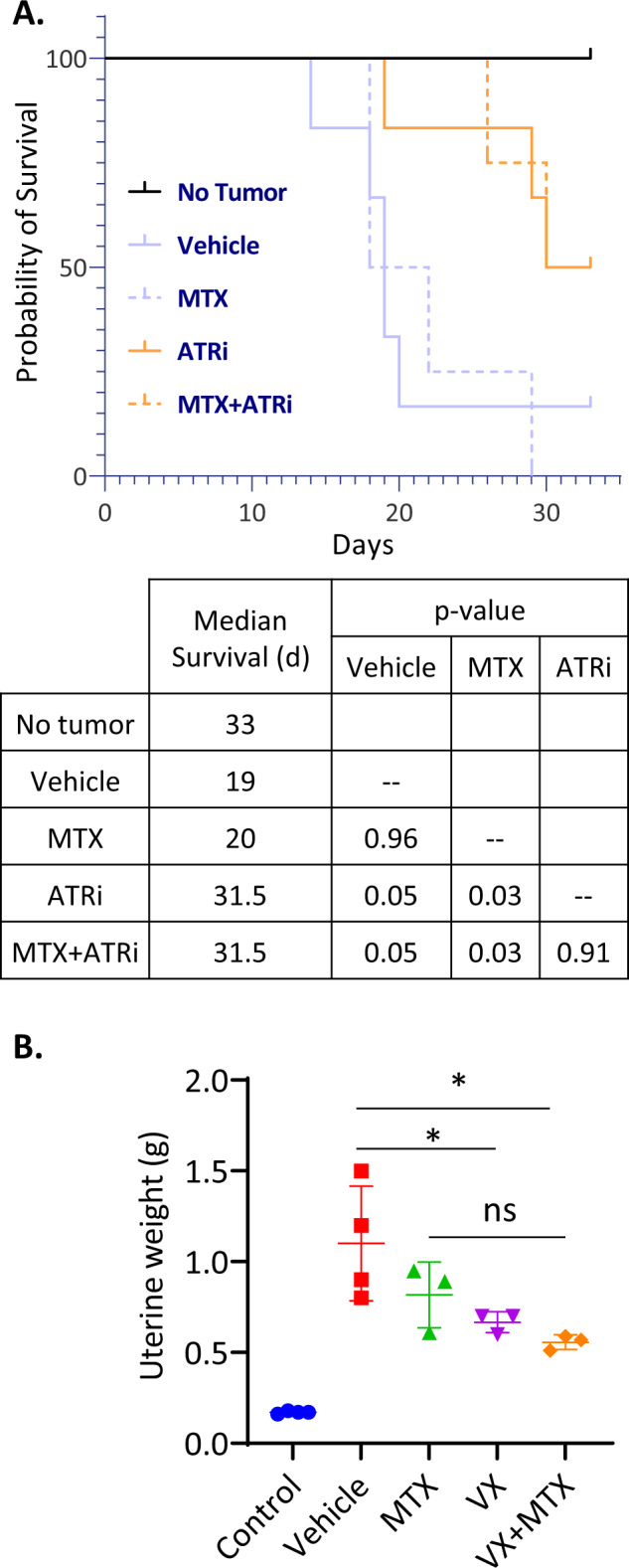
ATR inhibition significantly decreases tumour burden and prolongs survival in JEG3R mouse models. **A**–**B** JEG3R cells were injected at the uterine horn of *nude* mice. Mice received either Vehicle-only (VO), VX-970 (VX-60mg/kg) and/or Methotrexate (MTX-1mg/kg). **A** Kaplan–Meier curve (upper panel) and corresponding median survival and associated statistics (lower panel). **B** Uterine weight was determined at end-point. Statistics: (**A**–**B**) ANOVA. ***p < 0.005, **p < 0.01, *p < 0.05, ns not significant.

VX-970 is currently being tested in a variety of phase 1 and 2 clinical trials as single agent and in combination with various chemotherapeutics (www.clinicaltrials.org). Therefore, we tested if combining MTX with VX-970 also showed improved response over MTX alone in our in vivo resistance model. This demonstrated that the ATR inhibitor, VX-970, used as single agent, was more potent than MTX at promoting the overall survival of tumour-bearing mice (Fig. 7A). Indeed, 50% of the animals were still alive at Day 30 in the VX-970-treated group while all MTX-treated animals were dead by Day 29. However, while there was a trend towards improved response to the combination of MTX and VX-970 as compared to either agent alone, this was not statistically significant (Fig. 7A-B). Hence, our results suggest that ATR inhibition could be efficient as a monotherapy for the treatment of MTX-resistant choriocarcinoma.

## Discussion

Choriocarcinoma is a mostly curable disease, but achieving this involves using toxic combination chemotherapy in a large number of patients resistant to the standard-of-care MTX or ACT-D monotherapies [2]. Hence, novel strategies that can re-sensitise tumours to these agents, or replace combination therapies with less toxic alternatives, would significantly improve the outcome for patients with resistant disease. Here, we have identified two therapeutic agents, already used in clinical settings, as potential alternatives to the use of combination therapy: the CDK4/6 and ATR inhibitors Palbociclib and VX-970 (Berzosertib), respectively. These compounds provided significant growth inhibition in our MTX-R in vivo models (Figs. 5D–E, 7A). Palbociclib has been widely investigated and is well tolerated apart from some myelosuppression which can be easily managed [54]. Similarly, early results with Berzosertib also indicate that this agent is well tolerated and active in several cancer types [55]. Importantly, these agents are much less toxic than EMA/CO or other combination therapies with neither agent causing hair loss or much in the way of other classical chemotherapy toxicities. While Palbociclib did not sensitise JEG3R cells to MTX in vitro (Fig. 5C), VX-970 did promote MTX response in a synergistic manner (Fig. 6F-G). This result was mirrored by MTX sensitisation of JEG3R cells following siRNA silencing or CRISPR knockout of ATR, CHK1 or WEE1 (Fig. 6A-B and Supplementary Fig. 4B), suggesting that activation of the ATR pathway bears some responsibility for the emergence of MTX resistance. In line with this, we found that proteins of the ATR pathway were either overexpressed or hyperactivated in resistant versus sensitive cells (Fig. 4E-F). This was associated with increased DNA repair abilities, especially through NHEJ, which was increased in JEG3R in an ATR-dependent manner (Fig. 4H). This involvement of ATR in NHEJ may appear puzzling in view of the prominent role of this kinase in replication induced-stress management and HR [56, 57] rather than NHEJ. However, ATR and ATM were previously shown to both be able to partake in NHEJ depending on the configuration of the DNA damage and that ATR can significantly regulate NHEJ in cases where ATM activity is impaired [58], which appears to be the situation in JEG3R cells as summarised in Fig. 3F. Decrease in ATM activity was consistent with the decreased phosphorylation of E2F1 on S364 (Supplementary Fig. 1E), a target of CHK2, which was under-expressed in JEG3R cells (Fig. 4F). Unfortunately, the in vitro sensitisation to MTX was not observed in vivo (Fig. 7A), suggesting that either the concentrations of both inhibitors achieved at the tumour site were not sufficient to observe synergistic interaction or that additional tumour microenvironmental cues cancel out this effect. Also, it is worth noting that ATR inhibition with VX-970 was less effective than ATR knockout in potentiating the toxicity of methotrexate in vitro. This difference may be due to the incomplete inhibition of ATR activity by VX-970, or maybe associated with kinase-independent effects of ATR. Indeed, ATR was shown to have direct kinase-independent antiapoptotic effects through localisation at the mitochondria [59], and this may account for the difference observed. In addition to this, ATR interacts with a number of other proteins involved in the regulation of DNA repair, such as TOPBP1 and ATRIP [56], and these interactions could be more readily disrupted by knockout of ATR than its inhibition. In addition to ATR itself, our results show that inhibition of other kinases on the ATR pathway, CHK1 and WEE1, may also be promising avenues for further investigation (Fig. 6F). Indeed, the CHK1 and WEE1 inhibitors used in this study, LY-2603618 and MK-1775, have already demonstrated activity in clinical trials with response biomarkers similar to those seen modulated in our cell system and limited toxicity [60, 61]. The consistent response observed in our system by targeting several members of the ATR pathway may in itself be of clinical value. Indeed, acquired resistance to targeted therapy often involves mutation of the compounds’ target that prevents efficient compound-target interaction, as seen in many cancer types [62–65]. Hence, the ability to target another kinase on the same pathway once resistance to the initial compound has been acquired could provide successive lines of treatment and extend the therapeutic benefit to patients.

The main limitation of our study is that our results are limited to the only existing MTX sensitive/resistant choriocarcinoma cell line pair. Hence, validation of our results in newly established cell line pairs in the future will be essential to determining how widely applicable our findings are to MTX resistance in this disease. Also, future work on MTX sensitive/resistant cell line pairs in other cancers may provide evidence for wider relevance of our data to mechanisms of resistance to this chemotherapeutic agent. However, several lines of evidence suggest that our experimental model is of disease relevance and that the new therapeutic strategies proposed here may be applicable to MTX resistance in general. We have demonstrated that a type I interferon signature was associated with MTX resistance in the JEG3R cell line [66]. This signature has now been identified in several different malignancies and is more properly understood to represent cGAS-STING-STAT pathway activation [67]. It is now understood that STING pathway activation sensitises cells to immune checkpoint inhibitors, which have demonstrated very good activity in chemo-refractory choriocarcinoma [68]. The fact that STING activation also occurs with ATR inhibitors [69] further suggests that ATR inhibitors may be especially useful in resistant choriocarcinoma as well. There are limited data on molecular pathways involved in resistance to methotrexate from tissue samples. Bolze et al. assessed 34 cases of chemoresistance and identified activation of IFNγ in mono-chemoresistant choriocarcinoma and inhibition of IL2 and TNF in poly-chemoresistant choriocarcinoma [70]. In ovarian cancer, PARP-inhibitors activate interferon signalling, leading to clinical trials which combine PARP-inhibitors with immune checkpoint blockade [71]. As noted above, immune checkpoint blockade has demonstrated clinical activity in choriocarcinoma, so a similar strategy combining ATR inhibitors with immune checkpoint inhibitors may be active in choriocarcinoma. On the other hand, existing data suggest that Palbociclib may be of clinical use in the treatment of choriocarcinoma patients. Indeed, in another study on patient samples, we examined miRNA expression in post-molar GTN [72]. Increases in miRNAs linked to down regulation of *BLC2* and loss of BLC2 protein were both associated with malignant progression. Dual targeting of BCL2 and CDK4/6 has shown good activity in ER + breast cancer [73], and so choriocarcinoma patients with intrinsically lower tumour expression for BCL2 may be particularly responsive to treatment with Palbociclib.

Our study also highlighted the power of SILAC-based proteomic comparison of sensitive and resistant cell lines coupled with functional network building in highlighting therapeutically actionable pathways associated with resistance and the underlying molecular mechanisms involved. As such, these results may still provide cues to additional therapeutic compounds that could be tested to alleviate MTX-resistant choriocarcinoma. In particular, our results suggest that changes in metabolic processes and mRNA splicing are associated with MTX-resistance in choriocarcinoma (Fig. 1D-E). Indeed, changes to the mRNA splicing for folylpolyglutamate synthetase (FPGS), the enzyme that polyglutamates MTX to achieve the drug’s intracellular retention, was recently associated with reduced responsiveness to MTX treatment [74]. More generally, mRNA splicing has been found to impact response to therapy in cancer [75], making this proposed avenue of research topical. Similarly, we and others have reported how changes in cancer cell metabolism can underlie the acquisition of resistance to cancer therapy [76, 77], and this may be even more relevant in the case of MTX, which acts as an antimetabolite of the folate pathway. Finally, our analysis of publicly-available microarray datasets for MTX-sensitive and resistant osteosarcoma, breast, colon and pancreatic cancer cell lines suggests that some of the cell cycle changes that we report for choriocarcinoma may also be associated with MTX resistance in these other malignancies. Hence, Palbociclib may offer benefit in other drug-resistant cancers where MTX plays an important role in therapy including osteosarcomas [78], germ cell tumours [79] and in the management of leptomeningeal spread of cancers such as breast cancer [80].

In conclusion, our research has identified two new therapeutic approaches for the treatment of MTX-resistant choriocarcinoma that now warrant further investigation in patients with this cancer.

## Material and methods

### Cell culture

The human choriocarcinoma MTX-sensitive JEG3, and resistant JEG3R cell lines were a kind gift from Dr Kevin Elias (Boston, MA, USA). The JEG3 cell line was isolated from the Woods strain of the Erwin-Turner tumour by Kohler and colleagues [81]. The MTX-resistant (JEG3R) cell line was generated by Dr Kevin Elias through long-term exposure of JEG3 cells to increasing concentrations of MTX over a 14-month period [66]. The 3 A sub E placental cell line (thereafter referred as PLC) was purchased from ATCC. ATR-knockout JEG3 and JEG3R CRISPR cell lines were generated through clonal isolation of cells populations infected with guide RNA-encoding lentivirus particles produced in HEK293T cells following transfection with lentiCRISPR v.2 (Addgene plasmid # 52961), psPAX2 (packaging plasmid encoding HIV gag, pol, rev, and tat; Addgene plasmids # 12260) and MD2.G (encoding VSV-G; Addgene # 12259) plasmids. Sequences for guide RNA (gRNA) spacers used during this study were obtained from the GeCKOv2 Human Library B, which was produced by the lab of Feng Zheng [82]. gRNA sequences used for ATR was GGATCATGGAAGCCAGCTCC. All cell lines were cultured in Dulbecco’s Modified Eagle’s Medium (DMEM) supplemented with 10% (v/v) Foetal calf serum (FCS), 2mM L-glutamine, 50 units/ml Penicillin and 50 μg/ml streptomycin (complete DMEM). JEG3R cells were cultured with MTX for 72 h every fortnight to maintain MTX resistance. All cell lines were incubated in a humidified atmosphere of 10% CO2 at 37 °C.

### Kinome siRNA screen

The human kinome library V2.0 targeting 691 kinases with four individual oligonucleotides per target was obtained from Qiagen. Cells were transfected with 20 nM siRNA in 96-well plates in OptiMEM (ThermoFisher) using Lipofectamine RNAiMAX (ThermoFisher) according to manufacturer’s protocol. 48 h later, cells were treated with MTX (80 nM and 36 μM for the JEG-3 and JEG-3R cells, respectively) for 72 h prior to crystal violet staining.

### Plasmid DNA transfection

Cells were transfected with Lipofectamine 3000 (Invitrogen) following the manufacturer’s instructions and used for experiments 24 h later when expression is maximal. The pE2F1-Luc was a kind gift from Dr William Kaelin (Addgene). The HR and NHEJ DNA repair vectors were a kind gift from Georgios Giamas, University of Sussex, UK: pimEJ5GFP (NHEJ) and pDRGFP (HR).

### siRNA transfection

20 nM siRNA oligonucleotides (Dharmacon) were transfected using Lipofectamine 3000 (ThermoFisher) according to the manufacturer’s instructions. The siGENOME Non-targeting siRNA Pool #2 (Dharmacon) was used as non-targeted control.

### Proteomic profiling by SILAC-based mass-spectrometry

JEG3 cells were SILAC-labelled in DMEM-15 (13C615N4 -Arg, 13C615N2 – Lys), while JEG3R cells were cultured in DMEM-14 (unlabelled –Arg, –Lys) for at least ten cellular divisions. All media were supplemented with 2mM L-glutamine, 50 units/ml Penicillin and 50 μg/ml streptomycin and 10% dialysed FCS. Cells were harvested in SDT-lysis buffer (4% (w/v) SDS, 100 mM Tris/HCl pH 7.6, 0.1 M DTT) and heated at 95 °C for 5 min. The DNA was sheared by sonication and the lysates were centrifuged at 13,000 g for 10 min. Equal protein amounts from JEG3 and JEG3R cells were combined at 1:1 ratio prior to protein digestion.

Total proteomics profiling: Samples were reduced in 10 mM DTT and alkylated in 50 mM iodoacetamide prior to boiling in loading buffer 4X NuPAGE LDS (ThermoFisher). Protein mixtures were separated by SDS/PAGE using 4–12% Bis-Tris Novex mini-gels and bands visualised by Coomassie staining. Gel lanes were split into ten slices prior to in-gel tryptic digestion. Tryptic peptides were extracted by 1% formic acid/acetonitrile, lyophilised in a speedvac and resuspended in 1% formic acid.

Phospho-proteomics profiling: The proteins were digested using the FASP method, as previously described [83], exchanging SDS for urea in a centrifugal ultrafiltration unit, followed by protein digestion and elution. Peptides separation into 45 fractions was achieved using hydrophilic interaction liquid chromatography and enriched for phospho-peptides with titanium oxide (TiO2) prior to MS/MS [84].

Trypsin-digested peptides were separated using an Ultimate 3000 RSLC nanoflow liquid chromatography (LC) system. The HPLC system was coupled to a LTQ-Orbitrap Velos via a nanoelectrospray ion source. Full-scan MS survey spectra were acquired in the Orbitrap after accumulation of 1,000,000 ions. The fifteen most intense peptide ions from the preview scan in the Orbitrap were fragmented by collision-induced dissociation in the LTQ after the accumulation of 10,000 ions. Precursor ion charge state screening was enabled, and all unassigned charge states, as well as, singly charged species were rejected [85]. Data were acquired using the XcaliburTM software. The raw mass spectrometric data files were collated into a single quantitated dataset using MaxQuant (version 1.2.2.5) with the Andromeda search engine software. Peptide ratios were calculated for each arginine- and/or lysine-containing peptide as the peak area of labelled divided by that of non-labelled arginine/lysine for each single-scan mass spectrum. Peptide ratios for all arginine- and lysine-containing peptides sequenced for each protein were averaged. Data were normalised using 1/median ratio value for each identified protein group per labelled sample.

### Ion torrent NGS protocol

Library generation followed the protocol described in the Ion AmpliSeq Library Kit 2.0 User Guide using the Ion AmpliSeq Cancer Hotspot Panel v2 primer pool, comprising 207 amplicons covering approximately 2800 COSMIC mutations from 50 oncogenes and tumour suppressor genes.

NGS runs were performed on 316v2 chips on an Ion Torrent PGM. Base calls, PCR duplicate removal and quality control analysis occurred on an Ion Torrent server using tools from the Ion Torrent Suite (v4.0-r76860). Sequencing reads were aligned to the reference human genome 19 (hg19) using the Torrent Mapping Alignment Programme (TMAP). Variant calling was performed using the Torrent Server variantCaller plugin (v5.0.2.1). The data is deposited in ArrayExpress under accession E-MTAB-11432.

### Cytoscape network building

Cytoscape bioinformatic analysis was used to reveal enriched pathways and biological processes within the compiled lists of proteins found to be differentially expressed or phosphorylated between our cell lines (± 1.5 fold change in Log2 ratio JEG3R/JEG3) by the MS analysis. The total proteomics results were processed using the Reactome FI plugin and linkers were introduced to facilitate network construction. The obtained functional interaction network was then clustered by Reactome FI to reveal 14 groups of proteins based on modularity calculation. These were subjected to gene ontology (GO) analysis under Reactome FI, which was validated using the BinGO plugin in Cytoscape. Selected subnetworks were further simplified to only conserve the minimal number of linkers required for network connectivity. Nodes were coloured using continuous red-blue mapping of the Log2 ratio in expression/phosphorylation changes.

### Protein stability determination using cycloheximide

Cells were incubated with cycloheximide (CHX) (20 µg/ml) for either 2, 4, 6, 8, 10, 12, 16, 20, 24 or 36 h. Cells were collected and lysates analysed by SDS-PAGE/Western blotting.

### Western blotting

Cellular proteins were extracted using a Radio immunoprecipitation assay buffer (RIPA) (50 mM Tris-Cl, pH 7.4, 0.1% SDS, 0.1% sodium deoxycholate, 2% Triton X-100, 150 mM NaCl, 2 mM EDTA, 5% Glycerol supplemented with protease inhibitors cocktail tablets (Roche Diagnostics), 10 mM β-Glycerophosphate, 1 mM sodium orthovanadate, 10 mM sodium fluoride). Equal protein amounts were diluted in 2x Laemmli buffer, boiled for 5 min and analysed by SDS-PAGE/Western blotting using the relevant primary antibodies and HRP-conjugated secondary antibodies. Immunoreactivity was revealed using Pierce ECL or SuperSignal substrates. Blots were visualised using the quantitative Fusion Solo Chemiluminescence Imager and image analysis was performed using FIJI.

### qRT-PCR validation of target down regulation

Total cellular mRNA was extracted using Purelink RNA kit (Invitrogen) and converted into cDNA using High Capacity Reverse Transcriptase kit (Applied Biosystems). qRT-PCR was performed using Fast SYBR green master mix (Applied Biosystems) with gene specific primers on ABI 7900 HT real-time PCR machine. HPRT and GAPDH were used as internal controls. Quantification used the ΔΔCt method. A list of primers used is shown in Supplementary Table 1.

### CFSE proliferation assay

Cells were resuspended at a concentration of 1 × 10^6^ cells/ml in 0.1% BSA/PBS and labelled with 10 μM CFSE for 5 min at 37 °C. Five times volume of ice cold DMEM was added to the cells for 5 min on ice in order for dye quenching followed by three washes with 1x DMEM to remove the excess dye before re-plating onto 6 cm dishes. At each point cells were harvested, washed once with 1x PBS and fixed with 4% paraformaldehyde at room temperature for 15 min. Finally, cells were washed three times with PBS, resuspended in 1 ml PBS and kept at 4 °C before flow cytometry analysis on a BD FACS Canto.

### Cell cycle analysis by propidium iodide staining

Cells were harvested, washed once with PBS and fixed using drop-wise addition of ice cold 70% ethanol under vortexing followed by 30 min incubation at 4 °C. Pellets were washed twice with PBS prior to addition of 50 μl of ribonuclease (100 μg/ml) (Sigma-Aldrich) for 15 min. Following addition of 50 μg/ml propidium iodide (Sigma-Aldrich) the DNA profile was acquired using flow cytometry on a BD FACS Canto.

### DNA damage repair assay

Cellular DNA damage repair efficiency was tested using homologous recombination (HR) and non-homologous end joining (NHEJ) plasmids (kind gift from Georgios Giamas, University of Sussex, UK), both with an interrupted GFP gene. A diagram of the method is presented in the corresponding Supplementary figure. Briefly, plasmids were linearised by I-SceI restriction enzyme as follows: 10 µg of reporter construct (NHEJ or HR), 10 µl of 10x CutSmart® Buffer (NEB), 5 µl of I-SceI (25 U) and ddH2O for a total of 100 µl. The digestion mix was left overnight at 37 °C. The digested plasmids were heated to 65 °C for 10 min to denature the restriction enzyme and purified using the QIAquick PCR Purification kit (QIAGEN). Seeded cells were transfected in OptiMEM using Lipofectamine 3000 as per the manufacturer’s protocol (Invitrogen) with either 500 ng of linearised HR or NHEJ plasmid, together with 250 ng of mCherry plasmid as internal transfection control. The following transfection controls were used for flow cytometry calibration; 250 ng of GFP plasmid alone, 250 ng of mCherryplasmid only and non-linearised HR or NHEJ (GFP-disrupted) plasmids co-transfected with the mCherry plasmid. 5 h after transfection, the culture medium was replaced with fresh complete DMEM. 72 h following transfection, cells were harvested as previously described and analysed by flow cytometry on a BD LSR II Flow Cytometer.

### E2F1 luciferase reporter assay

Cells were transfected with 1 µg of pE2F1-Luc construct (Firefly luciferase under the transcriptional control of E2F1 binding site) and 100 ng of pRL-CMV (Renilla-luciferase normalisation control vector). 24 h following transfection, both Firefly- and Renilla-luciferase activities were quantified using the Dual-Glo Luciferase Assay System (Promega), according to the manufacturer’s instructions. Luminescence was detected using a PHERAstar Plus plate reader. The measured luminescence from the Firefly luciferase activity was normalised to that of the Renilla luciferase.

### Crystal violet staining

Cells were stained in 0.5% w/v crystal violet solution for 15 min. Plates were washed in water and air-dried. Crystal violet-precipitates were solubilised in 10% (v/v) acetic acid (30 min, room temperature, gentle shaking) and absorbance measured at 595 nm.

### Clonogenic assay

10^3^ JEG3 cells or 2.10^3^ JEG3R cells were plated per well in 6-well plates and allowed to adhere overnight. Cells were transfected with CDK4/CDK6 or AllStars siRNAs every 72 h, as described above. Cells were cultured for 15 days prior to crystal violet staining. Following scanning of the stained wells, the total area covered by colonies was quantified in FIJI.

### Comet assay

Cells were pelleted at 800 g and resuspended in ice cold PBS at 1 × 10^5^ cells/ml. The cell samples were combined with pre-warmed 0.7% low melting agarose (LMA) in PBS at 1:10 ratio (v/v) and carefully mixed by pipetting. Using a multichannel micropipette, 20 µl of sample/well were immediately transferred onto the pre-warmed (37 °C) OxiSelect 96-Well Comet Slide (Cell Biolabs). The slide was then transferred to 4 °C for 15 min, protected from light to allow the agarose to set. The slide was then placed in 50–100 ml pre-chilled lysis buffer (100 mM EDTA-Na2, 2.5 M NaCl, 10 mM Tris-HCl, 250 mM NaOH, pH 10, with 1% v/v Triton X-100 and 10% DMSO added immediately before use) for 30–60 min at 4 °C in the dark. The buffer was carefully aspirated and replaced with a pre-chilled alkaline solution (300 mM NaOH, pH >13, 1 mM EDTA) for 30 min at 4 °C in the dark. The slide was then transferred to a BioRad horizontal electrophoresis chamber filled with cold alkaline electrophoresis solution (300 mM NaOH, pH >13, 1 mM EDTA) until it covered the slide. Voltage was applied for 15–30 min at 1 volt/cm to produce 300 mA. The slide was then transferred horizontally to three 2 min washes in pre-chilled ddH_2_O. Water was finally replaced with cold 70% Ethanol for 5 min. Once the agarose was completely dry, 50 µl/well of diluted SYTOX Green stain (1:500) were added for 15 min at room temperature. The comets were visualised using an EVOS microscope (ThermoFisher) and images analysed using the CASPLab software.

### E2F1 sequencing

DNA was extracted from 2.5 × 10^6^ JEG3 and JEG3R cells using the QiaAMP mini kit (Qiagen). Primers designed to amplify the coding sequences of *E2F1* using Primer 3 (version 0.4.0) were synthesised by Thermo Fisher. The targeted regions were amplified from 30 ng of DNA using Q5 Hot Start High-Fidelity or OneTaq Hot Start Quick-Load polymerases (New England Biolabs (NEB)) with 30 PCR cycles and conditions and annealing temperatures recommended by NEB. PCR products from JEG3 and JEG3R cells were purified using Exo-CIP (NEB) and dideoxy sequenced (Genewiz Ltd). Sequencing traces were analysed using Seqman (DNASTAR). Primers used were as follow: Exon 1; F: ATAGAAAGGTCAGTGGGATGCG, R: CACAGAGCAGCAAACAGGGA, Exons 2-3; F: AGGTCTCTTCTGGCCTCACTC, R: CCCGTTTCCCCAGCTATAAGAT, Exon 4; F: CCATCATTTGTTTATCCCGCCC, R: CAGCCTCTTGAAGCACTAGGAT, Exons 5-7; F: CTTGTGAGCTGTTGGAGTGAGT, R: GAGCATCTCTGGAAACCCTGG.

### Bioinformatics analysis of publically available data

GEO microarray datasets from several studies comparing the gene expression profiles of MTX-sensitive and resistant cell lines were downloaded from the NCBI GDS web-site: GSE16070, GSE16080, GSE16066, GDS3330, GSE16089, GSE16082. Only data obtained using JetSet probes [86] were analysed for Cyclin A2 (213226_at), Cyclin B1 (228729_at), Cyclin D1 (208712_at), Cyclin E1 (213523_at), CDK1 (203213_at), CDK2 (204252_at), CDK4 (202246_s_at), CDK6 (224847_at), p16 (207039_at), p21 (202284_s_at), p27 (209112_at), E2F1 (204947_at), E2F2 (228361_at), RB1 (203132_at). Data were analysed in R and plots generated using the *lattice* package.

### Animal experiments

Orthotopic xenografts: 6-week-old female NOD SCID mice (strain NOD.CB17-PrkdcSCID/NCrCrl) were used. The ventral surface of mice was shaved with clippers, and mice anaesthetised with inhaled isoflurane. Ophthalmic ointment was applied to the eyes, and the mouse was placed on a heated surgical board with an anaesthetic nose cone. The abdomen was prepped with povidone-iodine alternating with 70% isopropyl alcohol, and the mouse was sterilely draped. The incision line was infiltrated with 0.4 ml of a 1:1 solution of 0.1% lidocaine and 0.05% bupivacaine using a 30-gauge needle. A 1.5 cm midline vertical incision was then made from approximately 0.25 cm above the urethra extending cephalad. The subcutaneous tissues were dissected bluntly until the fascia was exposed, then the fascia and peritoneum were incised sharply in the midline. The peritoneal incision was extended cephalad 1 cm. A blunt probe was used to identify the uterus and elevate the left uterine horn. The uterine horn was then cannulated with a 27-gauge needle and injected with 250,000 cells/100 µl of a 1:1 mix of Matrigel (Corning) and JEG3R cell suspension in complete media. The uterine horn was then returned to the abdomen, and the fascia and muscle were closed with a running 4-0 polyglactin braided suture. The skin was closed with a running 4-0 poligelcaprone monofilament suture. The mice were then given subcutaneous meloxicam 1.5 mg/kg in sterile saline for post-operative analgesia and moved to a heated recovery cage until fully recovered from anaesthesia. 5–7 mice/treatment group were injected with tumour cells and three mice received no tumour (sham surgery). 24 h later, mice were given a second meloxicam injection.

Drug Treatments: Treatments began on post-operative day 4. Mice in the MTX group received 1 mg/kg MTX (Sigma-Aldrich) via tail vein injection weekly in 80 µl saline. Mice in the oral gavage groups received either 100 µl of vehicle alone, comprising 10% D-alpha-tocopheryl polyethylene glycol succinate (Sigma-Aldrich) dissolved in a 50:50 mixture of dimethyl sulfoxide and water, or 100 µl of vehicle containing 125 mg/kg of palbociclib (Sigma-Aldrich). Oral gavage was administered via a feeding needle. Mice received daily oral gavage on post-operative days 4–11, then every other day oral gavage for post-operative days 13–17.

β-hCG measurement: Blood was collected from the mice via the submandibular vein on post-operative days 4, 11 and 18 and centrifuged to obtain serum. Serum aliquots were analysed in duplicates using a Human beta hCG ELISA kit (Abcam Cat # ab108638) according to the manufacturer’s instructions. Sterile filtered 10% FBS in PBS was used as a diluent buffer.

Necropsy: At end-point, mice were euthanized by carbon dioxide inhalation and necropsies were performed. The uteri were photographed in situ.

5–8 animals per group were used for these experiments as determined based on a one-way ANOVA calculation to keep the degrees of freedom between 10 and 20. Animals were randomly allocated to treatment groups by a technician blinded to the experimental conditions. Animal experiments were at least performed twice.

### Antibodies

Anti Phospho-E2F1 (S364) and Mdm2 were from Abcam. Anti DHFR was from antibodies-online.com. Anti ATR (Ser428), ATM (Ser1981), Rb (Ser780), Wee1 (Ser642), p53 (Ser15), p53 (Ser392), ATR, H2AX, Rb, Caspase-8, Caspase-9, Caspase-3, Caspase-7, CDK1, CDK2, CHK1, CHK2, cMYC, Cyclin A2, Cyclin B1, Cyclin D1, Cyclin E, PARP1 were from Cell Signalling Technology. Anti γH2AX (Ser139) was from Milipore. Anti RAD51, MRE11, ATM, P14, CDK4, CDK6, E2F1, E2F2, E2F4, P16, P18, P21, P27, P53 were from Santa Cruz Biotechnology. Anti E2F1 (Ser337) was from ThermoFisher. Anti Vinculin, β-Actin and β-Tubulin were from Sigma-Aldrich.

## Supplementary information

Supplementary Figures and legends

E2F1 exons sequencing traces

Supplementary Table 1

Supplementary Table 2

Supplementary Table 3

SILAC based total proteomics MS-MS data

SILAC based phosphoproteomics MS-MS data

Kinome siRNA screen data
