# Correction: ATR and CDK4/6 inhibition target the growth of methotrexate-resistant choriocarcinoma

**DOI:** 10.1038/s41388-022-02354-2

**Published:** 2022-05-30

**Authors:** Marina Georgiou, Panagiota Ntavelou, William Stokes, Rajat Roy, Geoffrey J. Maher, Tsvetana Stoilova, Josephine A.M.Y. Choo, Callum P. Rakhit, Miguel Martins, Paul Ajuh, Neil Horowitz, Ross S. Berkowitz, Kevin Elias, Michael J. Seckl, Olivier E. Pardo

**Affiliations:** 1 Division of Cancer, Department of Surgery and Cancer, Imperial College, London, UK; 2 MRC Toxicology Unit, University of Cambridge, Cambridge, UK; 3 Gemini Biosciences, Liverpool, UK; 4 Division of Gynecologic Oncology, Department of Obstetrics and Gynecology, Brigham and Women’s Hospital, Dana Farber Cancer Institute, Harvard Medical School, Boston, MA USA

**Keywords:** Cancer therapeutic resistance, Gynaecological cancer

Correction to: *Oncogene* 10.1038/s41388-022-02251-8, published online 18 March 2022

In this article, Josephine A.M.Y. Choo at affiliation Division of Cancer, Department of Surgery and Cancer, Imperial College, London, UK. was missing from the author list.

The original article has been corrected.

