# Supplementary material for: ATR and CDK4/6 inhibition target the growth of methotrexate-resistant choriocarcinoma
Source: Oncogene. 2022 Mar 18;41(18):2540–54. doi: 10.1038/s41388-022-02251-8 (PMC9054653; doi:10.1038/s41388-022-02251-8)

Supplementary Table 1: Primers used for qPCR

Supplementary Table 2: Tables represent output from the GO analysis performed on the Cytoscape networks presented in Figure 1 under Reactome FI filtered for False Discovery Rates (FDR) ≤ 0.05. Top and bottom panel: GO biological processes terms associated with cell cycle regulation (top) and DNA damage (bottom).

Supplementary Table 3: Targeted sequencing of PLC, JEG3 and JEG3R cells was performed on an Ion PGM System using the AmpliSeq Cancer Hotspot Panel v2.

Supplementary Excel Spreadsheet 1: SILAC-based total proteomics MS-MS data

Supplementary Excel Spreadsheet 2: SILAC-based phosphoproteomics MS-MS data

Supplementary Excel Spreadsheet 3: MTX-sensitisation kinome siRNA screen data

Supplementary Figure 1: Functional interaction networks from quantitative phosphoproteomics changes between JEG3R and JEG3 cells and phenotypic validation. (A-B) A functional interactome network was built based on the SILAC/MS-based quantitative phosphoproteomics changes between JEG3 and JEG3R cells. Data were analysed under Cytoscape using the Reactome FI plugin followed by modularisation based on network connectivity and Gene Ontology (GO) analysis. Modules corresponding to Cell Cycle (A) and DNA damage and repair (B)-related GO biological processes terms are shown. (B) Nodes involved in various DNA damage repair pathways are indicated: DDR; DNA damage response, HR; homologous recombination, NER; nucleotide excision repair, NHEJ; non-homologous end-joining, MMR; mismatch repair. Nodes' colour: red indicates increased and blue decreased phosphorylation of the indicated protein in JEG3R over JEG3. Green nodes are linkers introduced during network building. **(C)** JEG3 and JEG3R cells were treated in the presence of cycloheximide for the indicated times and cell lysates analysed by Western blotting. Signals for E2F1 were analysed by optical densitometry and normalised to those for vinculin used as a loading control. Graph shows mean fold change from t=0 ± SEM of 4 biological replicates. **(D)** An E2F1 reporter plasmid was transfected in JEG3 and JEG3R cells and E2F1 activity compared between these two cell lines using a luciferase-based readout. Data are mean ± SEM from biological triplicates with n=3. **(E)** Lysates from JEG3 and JEG3R cells were analysed by Western blotting for the indicated targets (upper panel). Lower panel: Ratio of optical densitometry measurements for the indicated phospho-states of E2F1 normalised to total E2F1 levels in JEG3R vs JEG3 cells. Data shown are mean ± SEM of 3 biological repeats. **(F)** Representative distribution of fluorescence of JEG3 and JEG3R cells stained with CFSE and analysed by flow cytometry at 0, 24, 48, 72 and 96 hours. (C and D) Statistics: Student t-test. *; $p<0.05$, ****; $p<0.001$.

Supplementary Figure 2: NHEJ is increased in JEG3R as compared to JEG3 cells. (A-C) Cell lysates were analysed by Western blotting for the indicated proteins with detection of β-Tubulin, Vinculin or β-Actin used as loading control. Results shown are representative of n=3. **(D)** Schematics of the plasmid-based DNA damage repair reporter assay. Efficient repair results in GFP expression. **(E)** Representative flow cytometry readouts of the plasmid-based DNA damage repair reporter assays for HR (Left) and NHEJ (Right). mCherry is expressed through a co-transfected plasmid as a control for transfection. o /mCherry; negative control cells transfected with non-digested HR or NHEJ reporter + mCherry plasmids, Ø/mCherry cells transfected with the I-SceI linearized reporter + mCherry plasmids. **(F)** Representative pictures of the tunnel assay performed on JEG3 and JEG3R cells. Sidebars represent 50 µm. **(G)** JEG3 and JEG3R cells were subjected to a time-course treatment

with cycloheximide (20 µg/ml). Cell lysates were analysed by Western blotting for p53 and Lamin B as a loading control (Left panel). Right panel: Graph shown represents the mean ± SEM of the ratio of optical densitometry for p53 normalised to Lamin B performed on three replicate experiments using Image J.

Supplementary Figure 3: NOD SCID mice injected orthotopically with JEG3R cells were treated with/without MTX and Palbociclib. Uterine weight was determined at end-point. Statistics: Student t-test. Unlabelled; p>0.05, *; p<0.05, **; p<0.01.

Supplementary Figure 4: CHK1 knockout sensitises choriocarcinoma cells to MTX. (A) JEG3 and JEG3R cells were subjected to CRISPR-mediated knockout of CHK1. Western blot for CHK1 verifies efficient knockout, Detection of Vinculin was used as a loading control. (B) CHK1-knockout or untargeted choriocarcinoma cells were exposed to a dose range of MTX and cell viability determined 72h later using Crystal violet staining.

Supp Table 1

Target Name	Forward primer	Reverse primer
ATM	TGTTCCAGGACACGAAGGGAGA	CAGGGTTCTCAGCACTATGGGA
ATR	GGAGATTTCCTGAGCATGTTCGG	GGCTTCTTTACTCCAGACCAATC
CDK1	GGAAACCAGGAAGCCTAGCATC	GGATGATTCAGTGCCATTTTGCC
CDK4	GTCGGCTTCAGAGTTTCCAC	TGCAGTCCACATATGCAACA
CHK1	GTGTCAGAGTCTCCCAGTGGAT	GTTCTGGCTGAGAACTGGAGTAC
CHK2	TCGAAAGCCAGCTTTACCTC	TGATCAGTCAGTTTATCCTAAGGC
Cyclin A2	GTCACCACATACTATGGACATG	AAGTTTTCCTCTCAGCACTGAC
Cyclin B	GACCTGTGTCAGGCTTTCTCTG	GGTATTTTGGTCTGACTGCTTGC
Cyclin D1	CAATGACCCCGCACGATTTC	CATGGAGGGCGGATTGGAA
Cyclin E	TCTTTGTCAGGTGTGGGGA	GAAATGGCCAAAATCGACAG
DHFR	CATGGTCTGGATAGTTGGTGGC	GTGTCACTTTCAAAGTCTTGCATG
DNA-PKcs	GTCATTACTTGTGATGAGCTACTCC	TGGTTCTTGGGCACGAATGT
E2F1	GGACCTGGAAACTGACCATCAG	CAGTGAGGTCTCATAGCGTGAC
E2F2	CTCTCTGAGCTTCAAGCACCTG	CTTGACGGCAATCACTGTCTGC
HPRT	TGACACTGGCAAAACAATGCA	GGTCCTTTTCACCAGCAAGCT
KI67	GAAAGAGTGGCAACCTGCCTTC	GCACCAAGTTTTACTACATCTGCC
Ku70	GCAGTGTCACCTCTGTTGGA	TATGAGCTGGTTACTCGCTTCCT
Ku80	GCGTTGATTGGGACCGAGTA	CATGTTGGCTACTGCTCACTTTG
LIG3	ACGCTGTGCCAAACAAAGG	CGTCGAATGCCACAAAGTAGC
LIG4	CTGGAACTGTATTGCCTGCTT	TCTCGTTTAACTGGCCTCGG
MAGEA10	TGGCAGTGATCCTGCACGGTAT	AAGCCTCCTCATACCACAGTGG
MDM2	TGTTTGGCGTGCCAAGCTTCTC	CACAGATGTACCTGAGTCCGATG
MME	AGAAATGCTTTCCGCAAGGCC	AGCCTC CCCACAGCATTTTCC
MSH3	CGAATTCTGTCATCCTGCAC	CCTGCAGCCAGTAGAGCTG
MSH6	CAGGGGTAACCCTCCATCTT	CAGGGGTAACCCTCCATCTT
P14	GGTTCTCGCAGTACCA	TGTTCGCCTCAGTTTCCCA
P16	CAAGATCACGCAAAAACCTCTG	CGACCCTATACACGTTGAACTG
P21	CATGGGTTCTGACGGACAT	AGTCAGTTCCTTGTGGAGCC
P27	TAATTGGGGCTCCGGCTAACT	TGCAGGTCGCTTCCTTATTCC
P53	CCTCAGCATCTTATCCGAGTGG	TGGATGGTGGTACAGTCAGAGC
RB	CAGAAGGTCTGCCAACACCAAC	TTGAGCACACGGTCGCTGTTAC
XLF1	GAGTCCACGGGTACTTCAGG	GGGCCTGTCAACATCAACTT
XRCC4	GGACATCAAACAAGAAGGGGAAACT	AGCTGAAGCCAACCCAGAGA
Wee1	GATGTGCGACAGACTCCTCAAG	CTGGCTTCCATGTCTTCACCAC
ZCCHC9	CGGTTGCAAACTTTGTGGCTCTG	GTCTGCACTCATTCCCTTTGCC

Supp Table 2

Biological Process	ROPGS	Gene set	Module	P-value	FDR
mitotic cell cycle	0.0286	332	39	0	2.82E-14
cell division	0.0216	251	24	0	2.82E-14
mitosis	0.017	197	18	0	3.55E-10
positive regulation of ubiquitin-protein ligase activity involved in mitotic cell cycle	0.0046	54	11	0	1.08E-07
regulation of ubiquitin-protein ligase activity involved in mitotic cell cycle	0.0049	57	11	0	1.08E-07
anaphase-promoting complex-dependent proteasomal ubiquitin-dependent protein catabolic process	0.0052	60	11	0	1.23E-07
mitotic chromosome condensation	0.0009	11	6	0	2.42E-07
negative regulation of ubiquitin-protein ligase activity involved in mitotic cell cycle	0.0041	48	9	0	2.13E-06
G1/S transition of mitotic cell cycle	0.0096	112	12	0	2.73E-06
DNA damage response, signal transduction by p53 class mediator resulting in cell cycle arrest	0.0039	45	8	0	1.26E-05
cell cycle	0.0132	153	8	0	5.88E-04
G2/M transition of mitotic cell cycle	0.0097	113	6	0	7.92E-04
G1/S transition of mitotic cell cycle	0.0096	112	8	0	8.64E-04
cytokinesis	0.0029	34	4	0.0005	0.0074604
mitotic sister chromatid segregation	0.0014	16	3	0.0006	0.0090977
spindle checkpoint	0.0003	4	2	0.0008	0.0107037
G2/M transition of mitotic cell cycle	0.0097	113	6	0.0012	0.0140564
mitotic nuclear envelope reassembly	0.0004	5	2	0.0013	0.0140564
cytokinesis after mitosis	0.0018	21	3	0.0014	0.015568
cell cycle arrest	0.009	105	5	0.0009	0.0184551
mitotic sister chromatid cohesion	0.0005	6	2	0.0018	0.0201052
regulation of cell cycle	0.0074	86	5	0.0021	0.0209253
mitotic cell cycle spindle assembly checkpoint	0.0023	27	3	0.0029	0.0258851
mitotic cell cycle	0.0286	332	6	0.0114	0.0343454
positive regulation of cyclin-dependent protein kinase activity involved in G1/S	0.0004	5	2	0.0018	0.036602
positive regulation of neuroblast proliferation	0.0016	19	3	0.0018	0.036602
mitotic nuclear envelope disassembly	0.0029	34	3	0.0055	0.0381602
cell division	0.0216	251	5	0.0141	0.0424117
cell proliferation	0.0238	277	8	0.0083	0.0430146
regulation of metaphase plate congression	0.0001	1	1	0.0103	0.0430146
mitotic spindle elongation	0.0001	1	1	0.0103	0.0430146
signal transduction involved in mitotic cell cycle G1/S transition DNA damage checkpoint	0.0001	1	1	0.0103	0.0430146
regulation of neural precursor cell proliferation	0.0005	6	2	0.0026	0.0450461
mitotic cell cycle	0.0286	332	11	0.003	0.0478155
positive regulation of mitotic cell cycle spindle assembly checkpoint	0.0004	5	1	0.028	0.0498488
regulation of G2/M transition of mitotic cell cycle	0.0007	8	1	0.0444	0.0498488

Biological Process	ROPGS	Gene set	Module	P-value	FDR
DNA repair	0.0188	219	22	0	2.06E-13
mismatch repair	0.0019	22	6	0	1.17E-05
response to DNA damage stimulus	0.0118	137	11	0	1.57E-05
negative regulation of DNA recombination	0.0005	6	4	0	3.12E-05
double-strand break repair	0.0044	51	7	0	6.05E-05
intra-S DNA damage checkpoint	0.0005	6	3	0	0.001219118
nucleotide-excision repair	0.0045	52	5	0.0002	0.004907767
base-excision repair	0.0026	30	4	0.0003	0.00603519
nucleotide-excision repair, DNA damage removal	0.0013	15	3	0.0005	0.008092178
double-strand break repair via nonhomologous end joining	0.0013	15	3	0.0005	0.008092178
DNA damage response, signal transduction resulting in induction of apoptosis	0.0034	39	4	0.0008	0.00993733
nucleotide-excision repair, DNA incision	0.0003	4	2	0.0005	0.005070564
double-strand break repair via homologous recombination	0.0044	51	4	0.002	0.020335276
DNA damage checkpoint	0.0022	25	3	0.0023	0.022239606
transcription-coupled nucleotide-excision repair	0.0031	36	3	0.0032	0.022607191
DNA damage response, detection of DNA damage	0.0008	9	2	0.004	0.032241908
nucleotide-excision repair, DNA damage removal	0.0013	15	2	0.0067	0.040282618
nucleotide-excision repair, DNA duplex unwinding	0.0001	1	1	0.0081	0.040282618
nucleotide-excision repair, DNA damage recognition	0.0001	1	1	0.0103	0.043014624
signal transduction involved in mitotic cell cycle G1/S transition DNA damage checkpoint	0.0001	1	1	0.0103	0.043014624
nucleotide-excision repair, DNA gap filling	0.0013	15	2	0.0108	0.043014624
nucleotide-excision repair	0.0045	52	3	0.0089	0.044362544

Supp Table 3

Sample ID	Chrom	Position	Ref	Variant	Allele Call	Frequency	Allele Source	Allele Name	Gene ID	Original Coverage
Placenta	chr7	55249063	G	A	Heterozygous	43.2	Novel	---	EGFR	95
Placenta	chr4	1807894	G	A	Homozygous	100	Novel	---	FGFR3	344
Placenta	chr4	55141055	A	G	Homozygous	100	Novel	---	PDGFRA	460
Placenta	chr10	43613843	G	T	Homozygous	100	Novel	---	RET	878
Placenta	chr9	133747535	A	G	Heterozygous	19.4	Novel	---	ABL1	32
Placenta	chr5	112173899	C	T	Heterozygous	59.9	Novel	---	APC	2191
Placenta	chr4	1806188	A	C	Heterozygous	31.7	Novel	---	FGFR3	93
Placenta	chr4	55152040	C	T	Heterozygous	98	Hotspot	COSM22413	PDGFRA	739
Placenta	chr17	37881453	G	C	Heterozygous	15.9	Novel	---	ERBB2	150
Chemo Sensitive	chr9	139399409	CAC	-	Heterozygous	1.8	Hotspot	COSM13047	NOTCH1	920
Chemo Sensitive	chr7	55249063	G	A	Homozygous	100	Novel	---	EGFR	651
Chemo Sensitive	chr4	1807894	G	A	Homozygous	100	Novel	---	FGFR3	971
Chemo Sensitive	chr4	55141055	A	G	Homozygous	100	Novel	---	PDGFRA	1632
Chemo Sensitive	chr10	43613843	G	T	Homozygous	100	Novel	---	RET	2233
Chemo Sensitive	chr7	116340262	A	G	Heterozygous	32.3	Hotspot	COSM710	MET	1217
Chemo Sensitive	chr7	116339672	C	T	Heterozygous	33.9	Novel	---	MET	4814
Chemo Sensitive	chr5	112175770	G	A	Heterozygous	50.2	Novel	---	APC	4481
Chemo Sensitive	chr2	29443733	A	T	Heterozygous	11.6	Novel	---	ALK	501
Chemo Sensitive	chr13	28608354	T	C	Heterozygous	5	Novel	---	FLT3	122
Chemo Sensitive	chr13	28608226	T	A	Heterozygous	25.3	Novel	---	FLT3	428
Chemo Sensitive	chr10	123279717	A	G	Heterozygous	88.9	Novel	---	FGFR2	127
Chemo Resistant	chr7	55249063	G	A	Homozygous	100	Novel	---	EGFR	235
Chemo Resistant	chr4	1807894	G	A	Homozygous	100	Novel	---	FGFR3	305
Chemo Resistant	chr4	55141055	A	G	Homozygous	100	Novel	---	PDGFRA	653
Chemo Resistant	chr10	43613843	G	T	Homozygous	100	Novel	---	RET	1013
Chemo Resistant	chr7	116340262	A	G	Heterozygous	29.1	Hotspot	COSM710	MET	546
Chemo Resistant	chr7	116339672	C	T	Heterozygous	32	Novel	---	MET	2061
Chemo Resistant	chr5	112175770	G	A	Heterozygous	40.3	Novel	---	APC	2040
Chemo Resistant	chr9	133747535	A	G	Heterozygous	16.2	Novel	---	ABL1	99

Colour Key
All
Both Cancers Samples
Placenta and Chemo Resistant
Unique to sample

Supp Figure 1

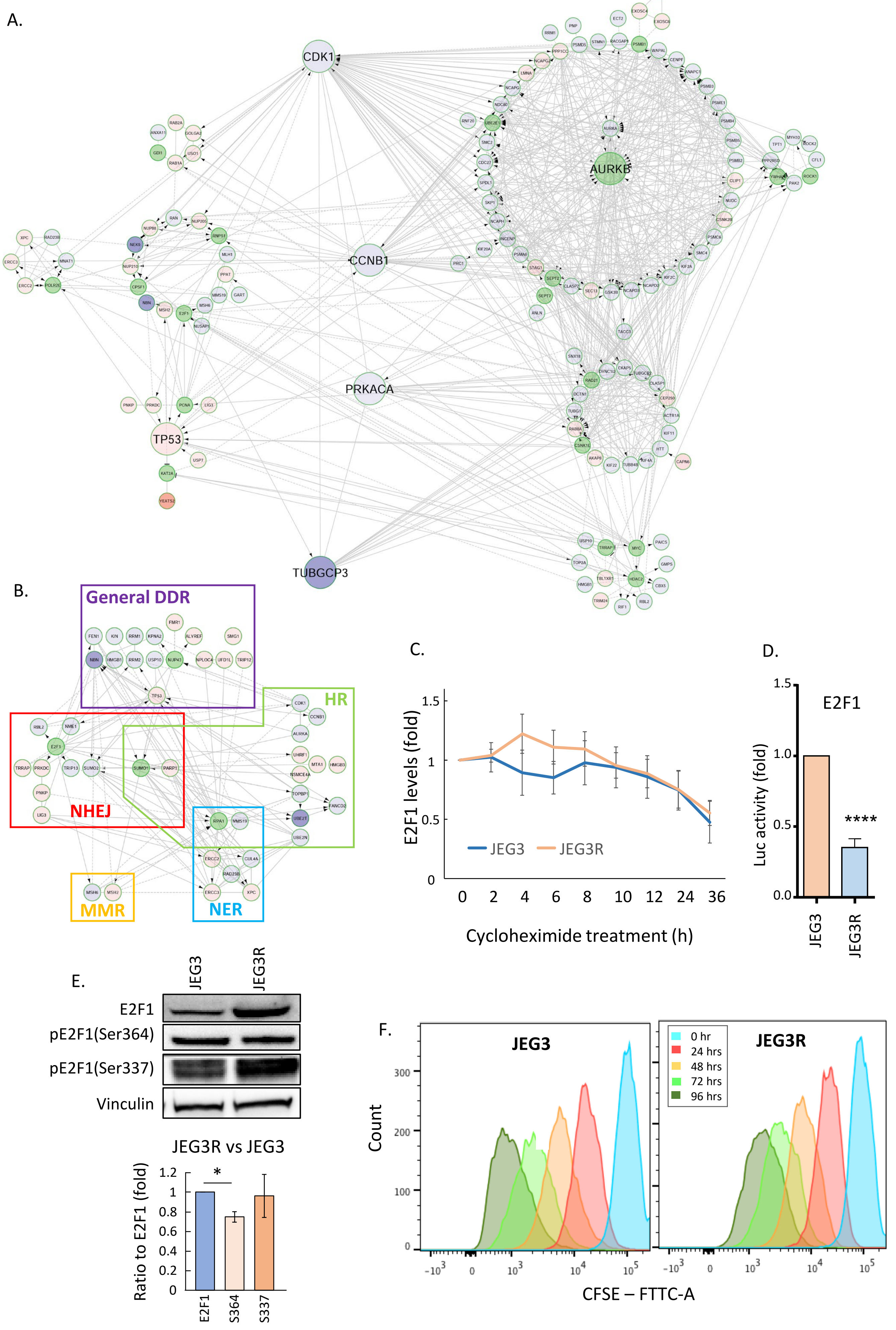

Supp Figure 2

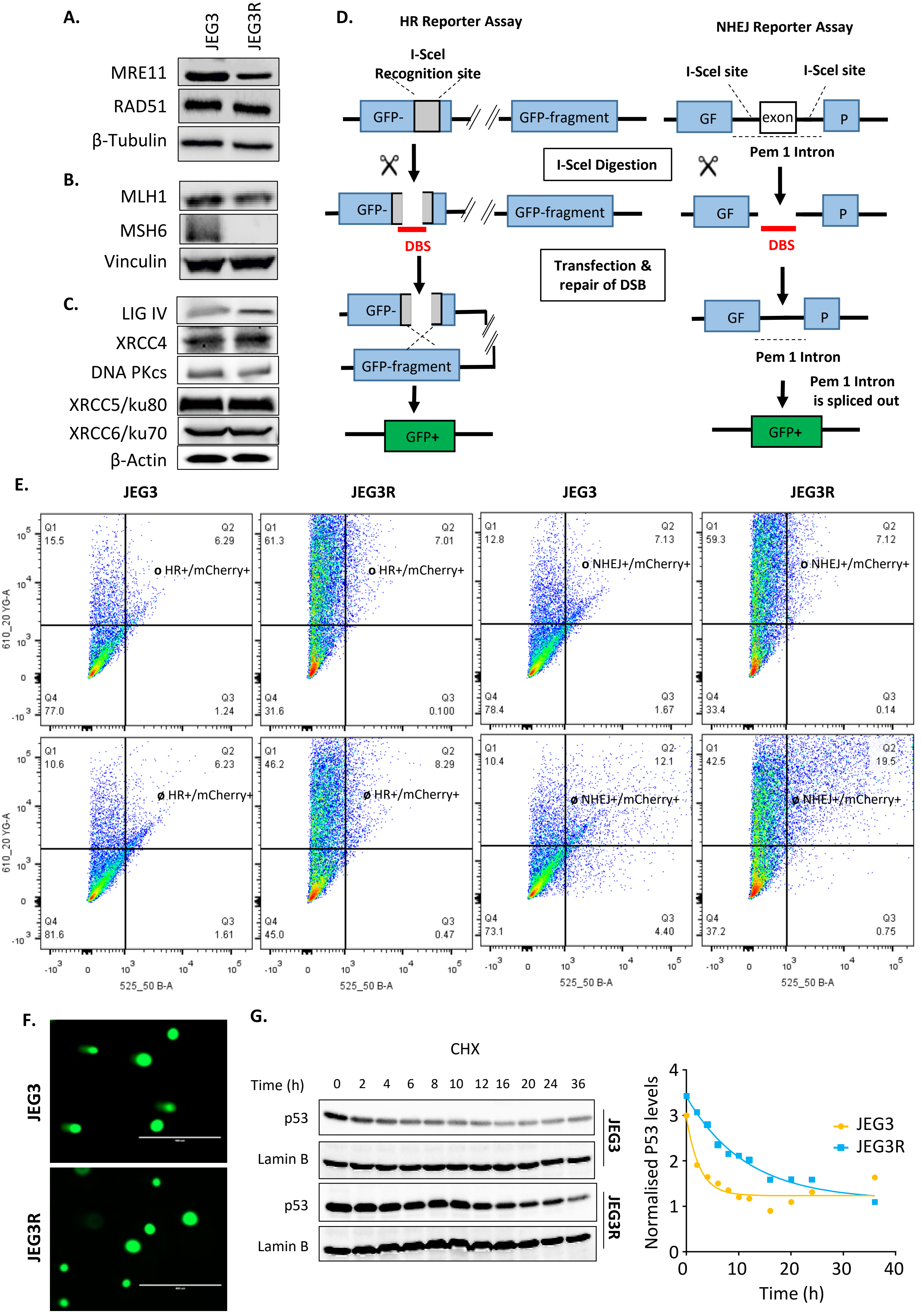

Supp Figure 3

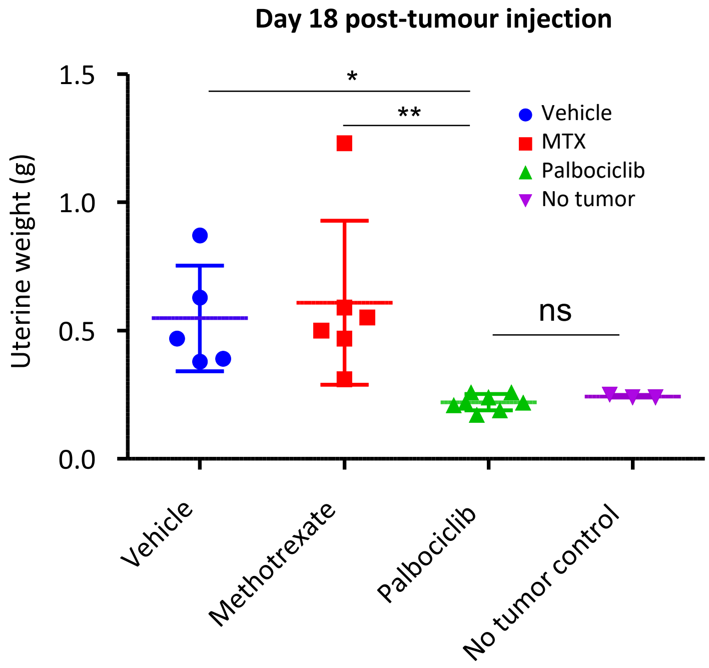

Supp Figure 4

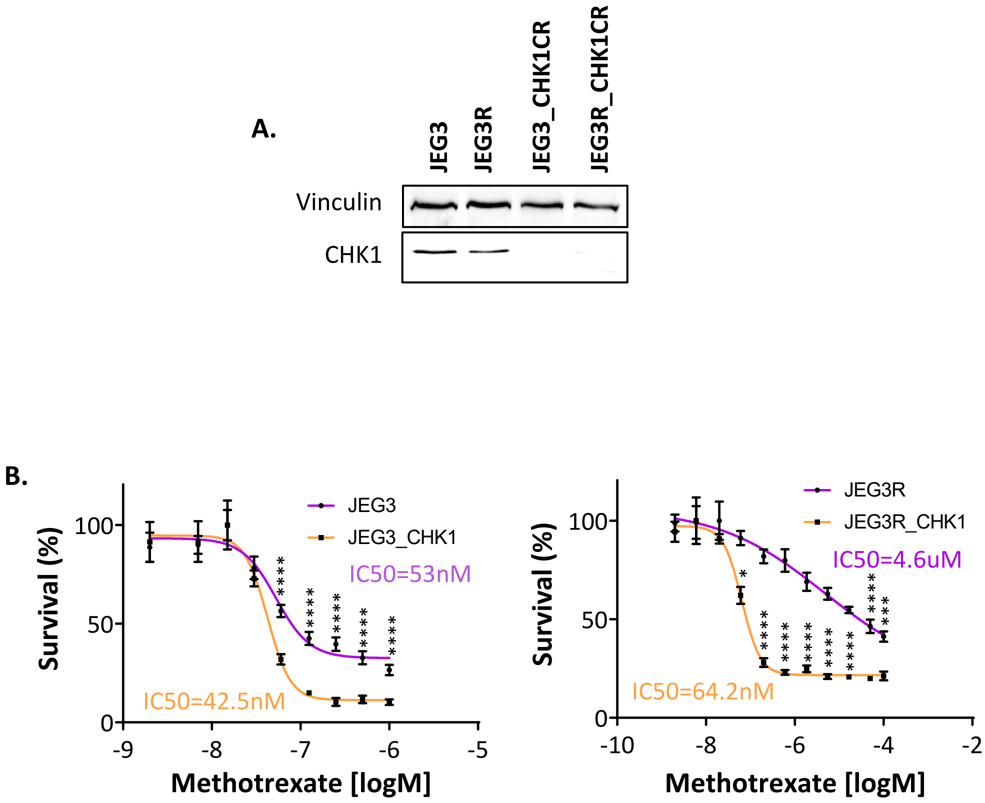

Supplement: Supplementary file 1 — Supplementary Figures and legends [file 41388_2022_2251_MOESM1_ESM.pdf]
