# Supplementary material for: ATR and CDK4/6 inhibition target the growth of methotrexate-resistant choriocarcinoma
Source: Oncogene. 2022 Mar 18;41(18):2540–54. doi: 10.1038/s41388-022-02251-8 (PMC9054653; doi:10.1038/s41388-022-02251-8)

Dideoxy sequencing traces for *E2F1* coding regions

Page	Trace
2	*E2F1* exon 1 Fw JEG3
3	*E2F1* exon 1 Fw JEG3R
4	*E2F1* exons 2-3 Fw JEG3
5	*E2F1* exons 2-3 Fw JEG3R
6	*E2F1* exon 4 Rev JEG3
7	*E2F1* exon 4 Rev JEG3R
8	*E2F1* exons 5-7 Fw JEG3
9	*E2F1* exons 5-7 Fw JEG3R

Traces were visualised using the Bioconductor package sangerseqR v1.22

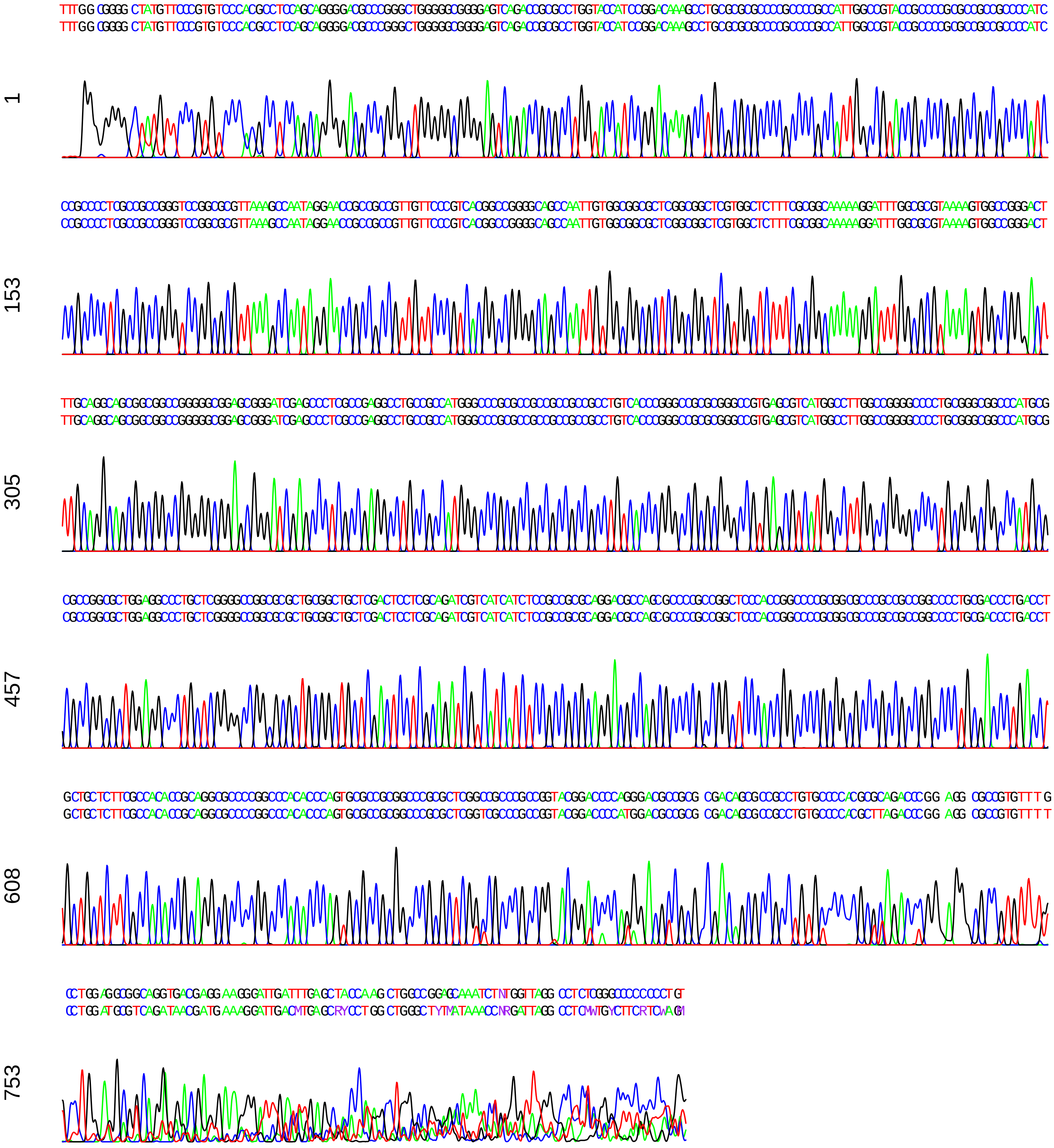
1
153
305
457
608
753

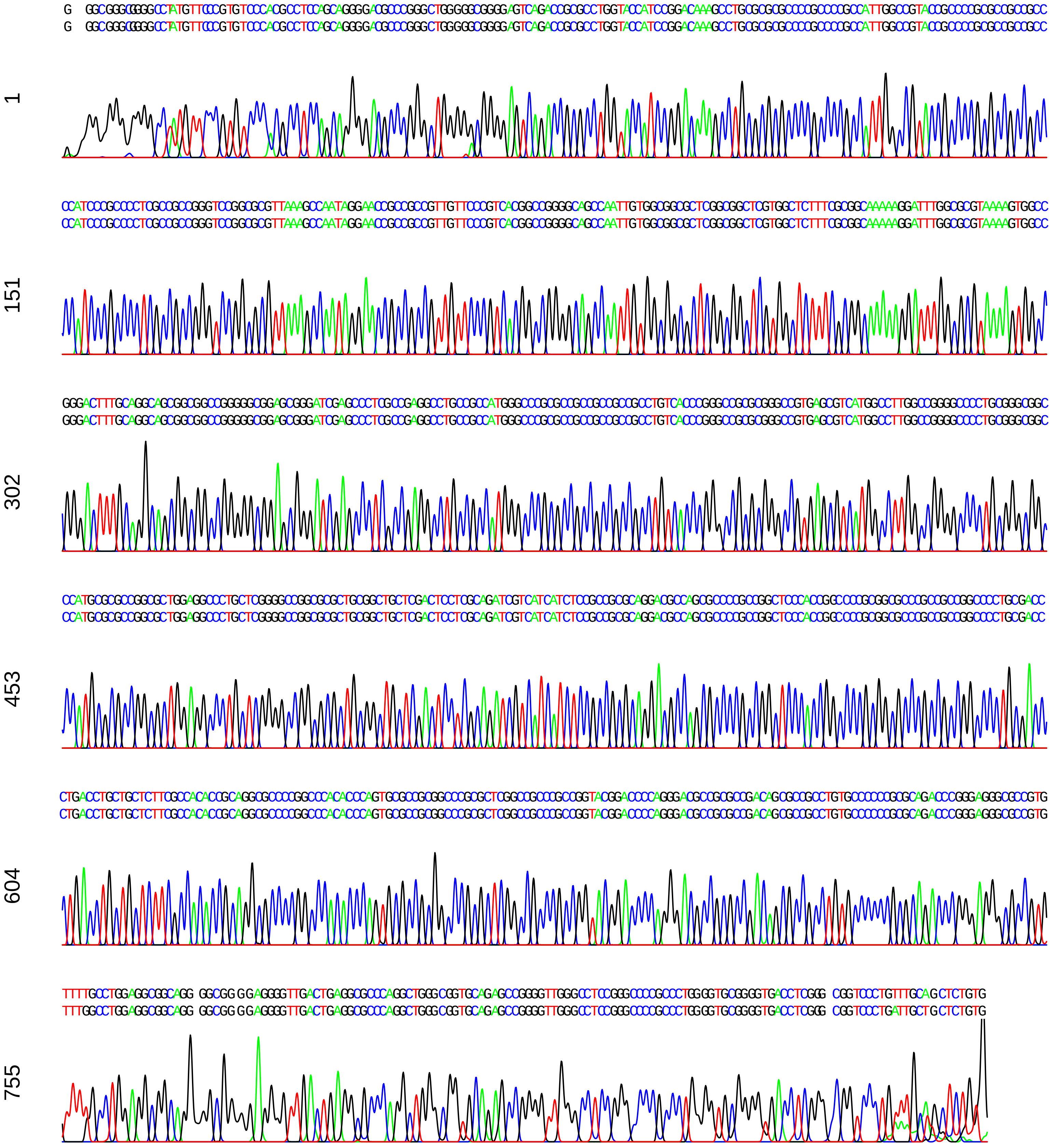
G GGCGGGCGGGGCCTATGTTCCCGTGTCCCACGCCTCCAGCAGGGGACGCCCGGGCTGGGGCGGGGAGTCAGACCGCGCCTGGTACCATCCGGACAAAGCCTGCGCGCGCCCCGCCCCGCCATTGGCCGTACCGCCCCGCGCCGCCGCC
G GGCGGGCGGGGCCTATGTTCCCGTGTCCCACGCCTCCAGCAGGGGACGCCCGGGCTGGGGCGGGGAGTCAGACCGCGCCTGGTACCATCCGGACAAAGCCTGCGCGCGCCCCGCCCCGCCATTGGCCGTACCGCCCCGCGCCGCCGCC
1
CCATCCCGCCCCTCGCCGCCGGGTCCGGCGCGTTAAAGCCAATAGGAACCGCCGCCGTTGTTCCCGTCACGGCCGGGGCAGCCAATTGTGGCGGCGCTCGGCGGCTCGTGGCTCTTTCGCGGCAAAAAGGATTTGGCGCGTAAAAGTGGCC
CCATCCCGCCCCTCGCCGCCGGGTCCGGCGCGTTAAAGCCAATAGGAACCGCCGCCGTTGTTCCCGTCACGGCCGGGGCAGCCAATTGTGGCGGCGCTCGGCGGCTCGTGGCTCTTTCGCGGCAAAAAGGATTTGGCGCGTAAAAGTGGCC
151
GGGACTTTGCAGGCAGCGGCGGCCGGGGGCGGAGCGGGATCGAGCCCTCGCCGAGGCCTGCCGCCATGGGCCCGCGCCGCCGCCGCCGCCTGTCACCCGGGCCGCGCGGGCCGTGAGCGTCATGGCCTTGGCCGGGGCCCCTGCGGGCGGC
GGGACTTTGCAGGCAGCGGCGGCCGGGGGCGGAGCGGGATCGAGCCCTCGCCGAGGCCTGCCGCCATGGGCCCGCGCCGCCGCCGCCGCCTGTCACCCGGGCCGCGCGGGCCGTGAGCGTCATGGCCTTGGCCGGGGCCCCTGCGGGCGGC
302
CCATGCGCGCCGGCGCTGGAGGCCCTGCTCGGGGCCGGCGCGCTGCGGCTGCTCGACTCCTCGCAGATCGTCATCATCTCCGCCGCGCAGGACGCCAGCGCCCCGCCGGCTCCCACCGGCCCCGCGGCGCCCGCCGCCGGCCCCTGCGACC
CCATGCGCGCCGGCGCTGGAGGCCCTGCTCGGGGCCGGCGCGCTGCGGCTGCTCGACTCCTCGCAGATCGTCATCATCTCCGCCGCGCAGGACGCCAGCGCCCCGCCGGCTCCCACCGGCCCCGCGGCGCCCGCCGCCGGCCCCTGCGACC
453
CTGACCTGCTGCTCTTCGCCACACCGCAGGCGCCCCGGCCCACACCCAGTGCGCCGCGGCCCGCGCTCGGCCGCCCGCCGGTACGGACCCCAGGGACGCCGCGCCGACAGCGCCGCCTGTGCCCCCCGCGCAGACCCGGGAGGGCGCCGTG
CTGACCTGCTGCTCTTCGCCACACCGCAGGCGCCCCGGCCCACACCCAGTGCGCCGCGGCCCGCGCTCGGCCGCCCGCCGGTACGGACCCCAGGGACGCCGCGCCGACAGCGCCGCCTGTGCCCCCCGCGCAGACCCGGGAGGGCGCCGTG
604
TTTTGCCTGGAGGCGGCAGG GGCGG GGAGGGGTTGACTGAGGCGCCCAGGCTGGGCGGTGCAGAGCCGGGGTTGGGCCTCCGGGCCCCGCCCTGGGTGCGGGGTGACCTCGGG CGGTCCCTGTTTGCAGCTCTGTG
TTTGGCCTGGAGGCGGCAGG GGCGG GGAGGGGTTGACTGAGGCGCCCAGGCTGGGCGGTGCAGAGCCGGGGTTGGGCCTCCGGGCCCCGCCCTGGGTGCGGGGTGACCTCGGG CGGTCCCTGATTGCTGCTCTGTG
755

TC CCCAGGGTGAAGCGGAGGCTGGACCTGGAAACTGACCATCAGTACCTGGCCGAGAGCAGTGGGCCAGCTCGGGGCAGAGGCCGCCATCCAGGAAAAGGTACCTATGGAGCTTGGGCTGGGCAGACCCCCTGTGACCAGGCCCAGG
TC CACAGGGTGAAGCGGAGGCTGGACCTGGAAACTGACCATCAGTACCTGGCCGAGAGCAGTGGGCCAGCTCGGGGCAGAGGCCGCCATCCAGGAAAAGGTACCTATGGAGCTTGGGCTGGGCAGACCCCCTGTGACCAGGCCCAGG

1

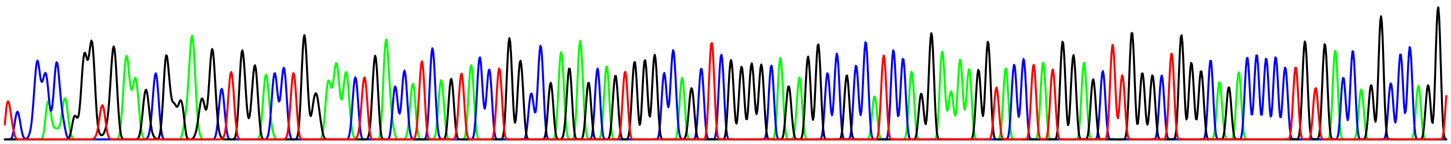

TTAGGGATGTGAACAGACAAACATGCTTGCAAGCTAACGTCTGGCTTGTCGGGCTTGAGCCAGGCCTCTGAGGACCCTGATGGGCAGTTGGAGTGGCCACCTGGCTTGGTTTCTCCAGGCCCTGAGGGGTGAGGAGTTCCCCAGCCAGAAGA
TTAGGGATGTGAACAGACAAACATGCTTGCAAGCTAACGTCTGGCTTGTCGGGCTTGAGCCAGGCCTCTGAGGACCCTGATGGGCAGTTGGAGTGGCCACCTGGCTTGGTTTCTCCAGGCCCTGAGGGGTGAGGAGTTCCCCAGCCAGAAGA

148

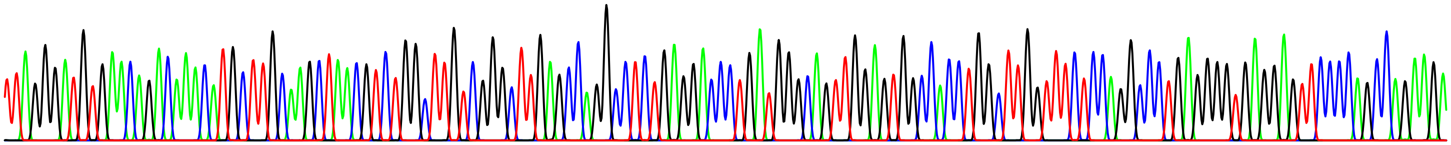

AGAATGCTGTGGTTGGCCTCGCCAAGGGGAAGGAGCTGGCTGTCCCCCAGCCCCCATCATCCTGCTGCCCTGCACTGCCAGAGTGGCAGGCCTGGCTGGAGGTGGGGGTGGATGCTGAAGGGCCCTGTCTTGCCTGACCACACCCCCACAGGT
AGAATGCTGTGGTTGGCCTCGCCAAGGGGAAGGAGCTGGCTGTCCCCCAGCCCCCATCATCCTGCTGCCCTGCACTGCCAGAGTGGCAGGCCTGGCTGGAGGTGGGGGTGGATGCTGAAGGGCCCTGTCTTGCCTGACCACACCCCCACAGGT

300

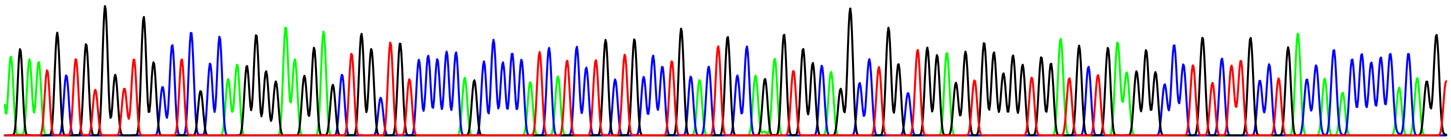

GTGAAATCCCCGGGGGAGAAGTCACGCTATGAGACCTCACTGAATCTGACCACCAAGCGCTTCCTGGAGCTGCTGAGCCACTCGGCTGACGGTGTCGTCGACCTGAACTGGGCTGCCGAGGTGCTGAAGGTGCAGAAGCGGCGCATCTATG
GTGAAATCCCCGGGGGAGAAGTCACGCTATGAGACCTCACTGAATCTGACCACCAAGCGCTTCCTGGAGCTGCTGAGCCACTCGGCTGACGGTGTCGTCGACCTGAACTGGGCTGCCGAGGTGCTGAAGGTGCAGAAGCGGCGCATCTATG

453

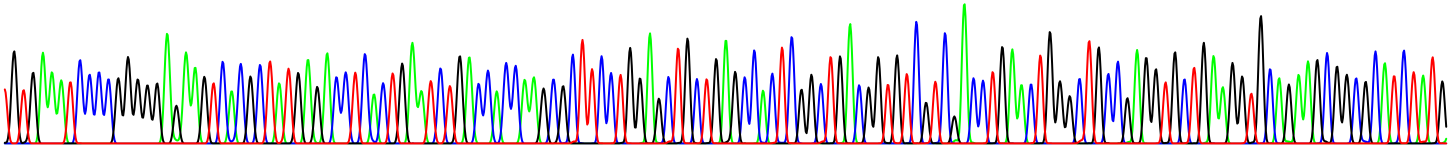

ACATCACCAACGTCCTTGAGGGCATCCAGCTCATTGCCAAGAAGTCCAAGAACCACATCCAGTGGCTGTAGGTACCGGCCACACAGGAGGGCAGGCACACCTGCCCATGCCAGCCTGGAGGAGCTGGTAGTAATACCCACTGTGGCTGC
ACATCACCAACGTCCTTGAGGGCATCCAGCTCATTGCCAAGAAGTCCAAGAACCACATCCAGTGGCTGTAGGTACCGGCCACACAGGAGGGCAGGCACACCTGCCCATGCCAGCCTGGAGGAGCTGGTAGTAATACCCACTGTGGCTGC

604

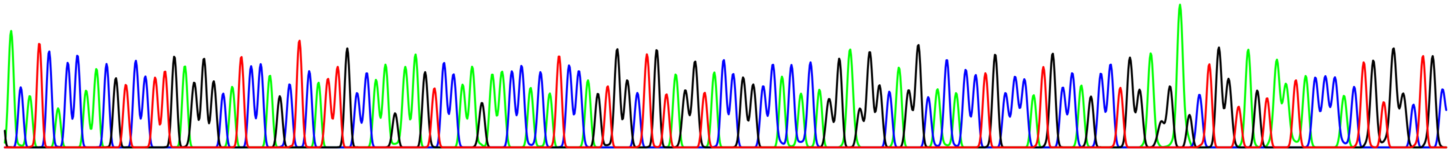

CTGCATCCCCATCTTATAGCTG GGAAAACGGG A A
CTGCATCCCCATCTTATAGCTG GGAAAACGGG A S

753

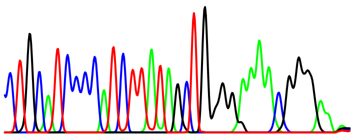

AGAGGGACGGCTTTCGCCCATGGTGAAGCGGAGGCTGGACCTGGAACTGACCATCAGTACCTGGCCGAGAGCAGTGGGCCAGCTCGGGGCAGAGGCCGCCATCCAGGAAAAGGTACCTATGGAGCTTGGGCTGGGCAGACCCCCTGTG
AGAGCCTCGGTTTTCGCCCATGGTGAAGCGGAGGCTGGACCTGGAACTGACCATCAGTACCTGGCCGAGAGCAGTGGGCCAGCTCGGGGCAGAGGCCGCCATCCAGGAAAAGGTACCTATGGAGCTTGGGCTGGGCAGACCCCCTGTG

1

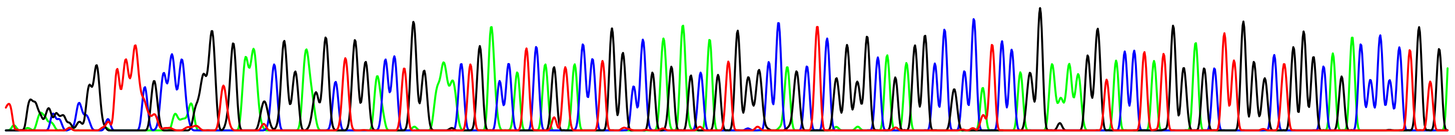

ACCAGGCCCAGGTTAGGGATGTGAACAGACAAACATGCTTGCAAGCTAACGTCTGGCTTGTCGGGCTTGAGCCAGGCCTCTGAGGACCCTGATGGGCAGTTGGAGTGGCCACCTGGCTTGGTTTCTCCAGGCCCTGAGGGGTGAGGAGTTC
ACCAGGCCCAGGTTAGGGATGTGAACAGACAAACATGCTTGCAAGCTAACGTCTGGCTTGTCGGGCTTGAGCCAGGCCTCTGAGGACCCTGATGGGCAGTTGGAGTGGCCACCTGGCTTGGTTTCTCCAGGCCCTGAGGGGTGAGGAGTTC

150

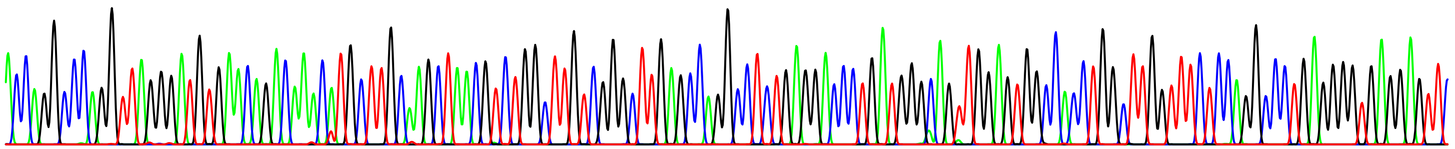

CCCAGCCAGAAGAAGAATGCTGTGGTTGGCCTCGCCAAGGGGAAGGAGCTGGCTGTCCCCCAGCCCCCATCATCCTGCTGCCCTGCACTGCCAGAGTGGCAGGCCTGGCTGGAGGTGGGGGTGGATGCTGAAGGGCCCTGTCTTGCCTGAC
CCCAGCCAGAAGAAGAATGCTGTGGTTGGCCTCGCCAAGGGGAAGGAGCTGGCTGTCCCCCAGCCCCCATCATCCTGCTGCCCTGCACTGCCAGAGTGGCAGGCCTGGCTGGAGGTGGGGGTGGATGCTGAAGGGCCCTGTCTTGCCTGAC

301

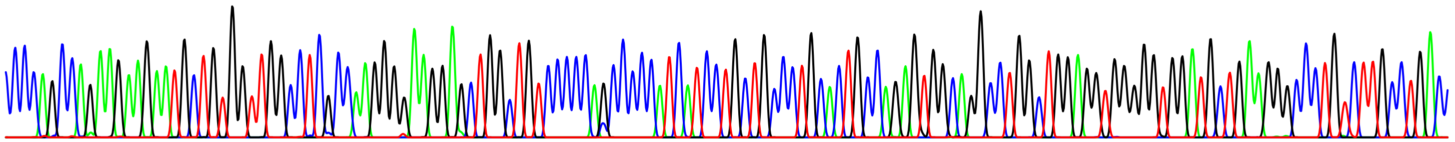

CACACCCCCACAGGTGTGAAATCCCCGGGGGAGAAGTCACGCTATGAGACCTCACTGAATCTGACCACCAAGCGGCTTCCTGGAGCTGCTGAGCCACTCGGCTGACGGTGTCGTCGACCTGAACTGGGCTGCCGAGGTGCTGAAGGTGCAGAA
CACACCCCCACAGGTGTGAAATCCCCGGGGGAGAAGTCACGCTATGAGACCTCACTGAATCTGACCACCAAGCGGCTTCCTGGAGCTGCTGAGCCACTCGGCTGACGGTGTCGTCGACCTGAACTGGGCTGCCGAGGTGCTGAAGGTGCAGAA

452

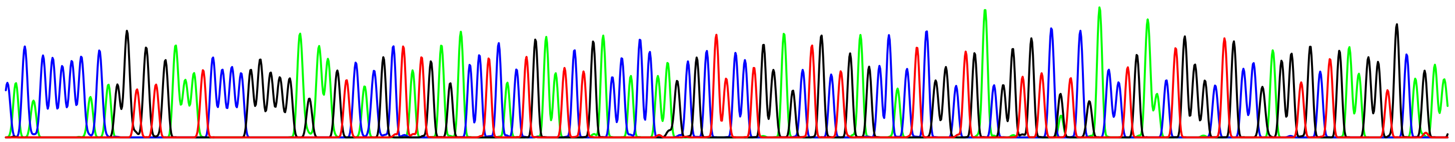

GCGGCGCATCTATGACATCACCAACGTCCTTGAGGGCATCCAGCTCATTGCCAAGAAGTCCAAGAACCACATCCAGTGGCTGTAGGTACCGGCCACACAGGAGGGCAGGCACACCTGCCCATGCCAGCCTGGAGGAGCTGGTAGTAATA
GCGGCGCATCTATGACATCACCAACGTCCTTGAGGGCATCCAGCTCATTGCCAAGAAGTCCAAGAACCACATCCAGTGGCTGTAGGTACCGGCCACACAGGAGGGCAGGCACACCTGCCCATGCCAGCCTGGAGGAGCTGGTAGTAATA

604

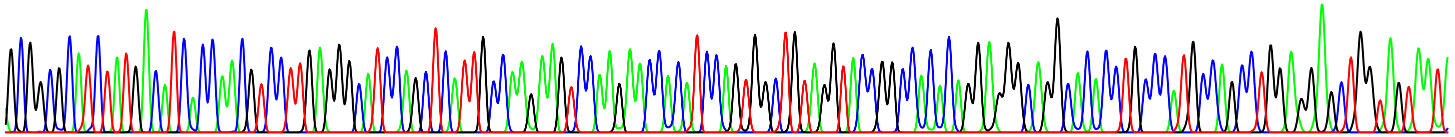

CCCACTGTGGCTGCCTGCATCCCCATCTTATAGCTGGGGAAACGGGAAN
CCCACTGTGGCTGCCTGCATCCCCATCTTATAGCTGGGGAAACGGGAAN

753

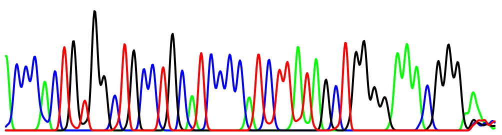

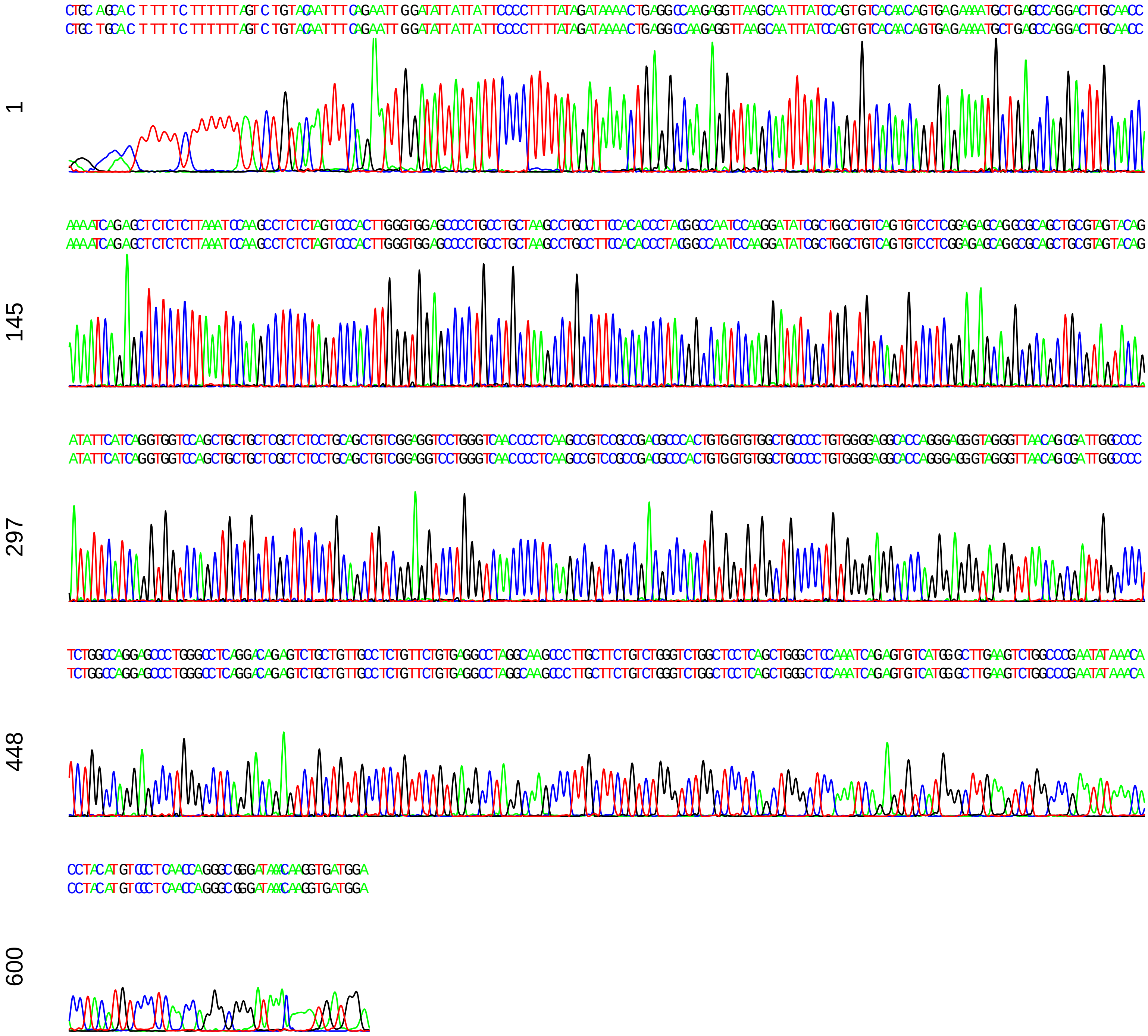
1
145
297
448
600

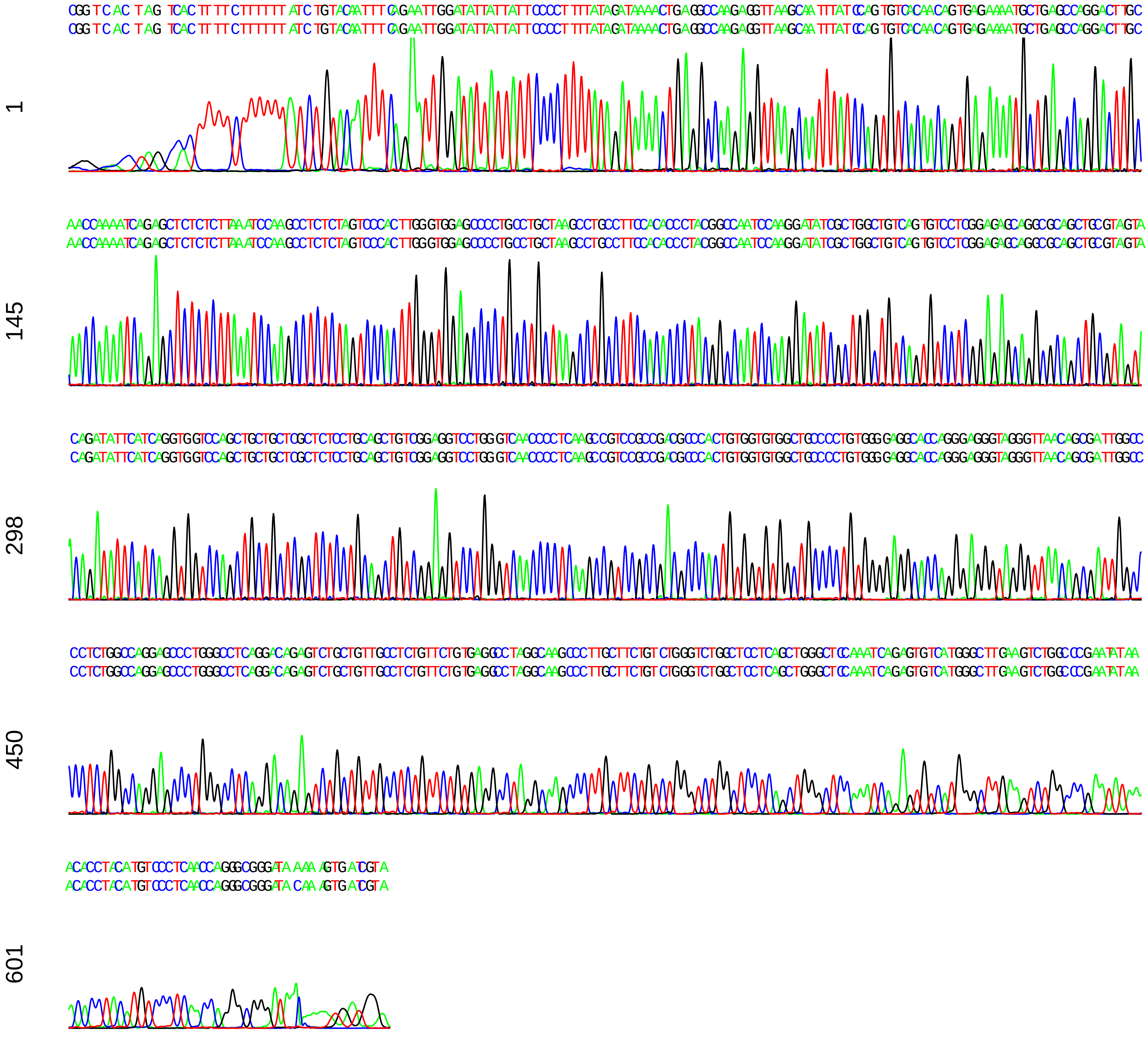
1
145
298
450
601

CCGAAGGTGGTATGTGGTCCTTGACTCTGCCACCCTGGCCTACGTGACGTGTCAGGACCTTCGTAGCATTGCAGACCCTGCAGAGCAGATGGTTATGGTGATCAAAGCCCCTCCTGAGACCCAGCTCCAAGCCGTGGACTCTTCGGAGGTG
CCGAAGGTGGTATGTGGTCCTTGACTCTGCCACCCTGGCCTACGTGACGTGTCAGGACCTTCGTAGCATTGCAGACCCTGCAGAGCAGATGGTTATGGTGATCAAAGCCCCTCCTGAGACCCAGCTCCAAGCCGTGGACTCTTCGGAGGTG

1

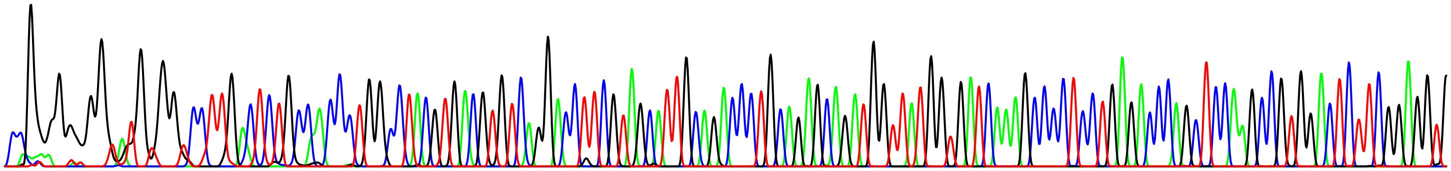

AGATCTGGGAACTCCGGGCCCAACTGGGCTGGGCTGGGCTGGGCTGGTGGTCACCTGGGCCTCAGTTTACCCTGCCTGCTGCTTCCACCCAGAACTTTCAGATCTCCCTTAAGAGCAAACAAGGCCCGATCGATGTTTTCCTGTGCCCTGA
AGATCTGGGAACTCCGGGCCCAACTGGGCTGGGCTGGGCTGGGCTGGTGGTCACCTGGGCCTCAGTTTACCCTGCCTGCTGCTTCCACCCAGAACTTTCAGATCTCCCTTAAGAGCAAACAAGGCCCGATCGATGTTTTCCTGTGCCCTGA

152

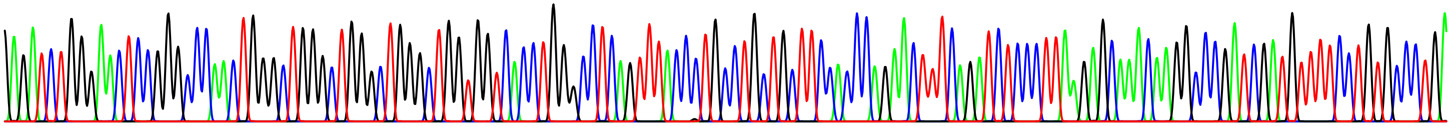

GGAGACCGTAGGTGGGATCAGCCCTGGGAAGACCCCATCCCAGGAGGTCACTTCTGAGGAGGAGAACAGGGCCACTGACTCTGCCACCATAGTGTCACCACCACCATCATCTCCCCCCTCATCCCTCACCACAGATCCCAGCCAGTCTCTAC
GGAGACCGTAGGTGGGATCAGCCCTGGGAAGACCCCATCCCAGGAGGTCACTTCTGAGGAGGAGAACAGGGCCACTGACTCTGCCACCATAGTGTCACCACCACCATCATCTCCCCCCTCATCCCTCACCACAGATCCCAGCCAGTCTCTAC

303

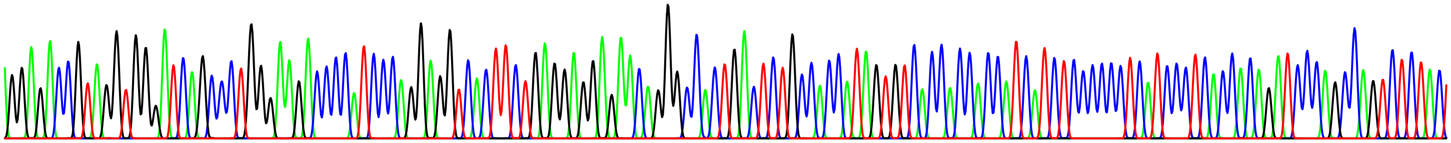

TCAGCCTGGAGCAAGGTGGGTGATGGGTAGGTGGGTGGGGTGGGGCAGGGCCCCTCTTCTGGGGGGTGGGCAGGCACGACAGCCCCTGCCTTCCTCCCTGCTGGGGCATCCCCGGCCTGTGATGCTCCCCGTCTCCCCAGAACCGCTGTTG
TCAGCCTGGAGCAAGGTGGGTGATGGGTAGGTGGGTGGGGTGGGGCAGGGCCCCTCTTCTGGGGGGTGGGCAGGCACGACAGCCCCTGCCTTCCTCCCTGCTGGGGCATCCCCGGCCTGTGATGCTCCCCGTCTCCCCAGAACCGCTGTTG

455

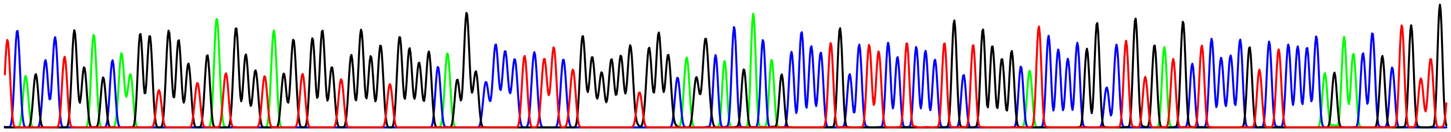

TCCCGGATGGGCAGCCTGCGGGCTCCCGTGGACGAGGACCGCCTGTCCCCGCTGGTGGCGGCCGACTCGCTCCTGGAGCATGTGCGGGAGGACTTCTCCGGCCTCCTCCCTGAGGAGTTCATCAGCCTTTCCCCACCCCACGAGGCCCT
TCCCGGATGGGCAGCCTGCGGGCTCCCGTGGACGAGGACCGCCTGTCCCCGCTGGTGGCGGCCGACTCGCTCCTGGAGCATGTGCGGGAGGACTTCTCCGGCCTCCTCCCTGAGGAGTTCATCAGCCTTTCCCCACCCCACGAGGCCCT

606

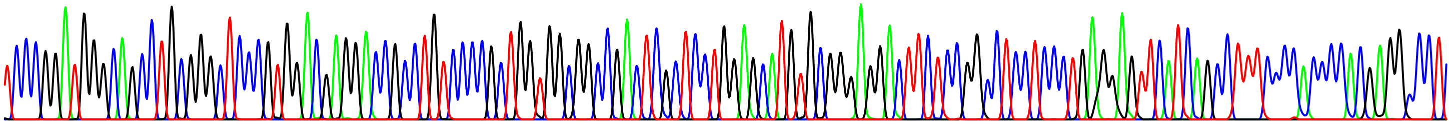

CGACTACCACTTCGGCCTCGAGGAGGGCGAGGGCATCAGAGACCTCTTCGACTGTGACTTTGGGGACCTCACC CCCTGGATTTCTGACAGGGCTTGGAGGGACCAGG TTTCAGAAGATGCTCAAT
CGACTACCACTTCGGCCTCGAGGAGGGCGAGGGCATCAGAGACCTCTTCGACTGTGACTTTGGGGACCTCACC CCCTGGATTTCTGACAGGGCTTGGAGGGACCAGG TTTCAGAAGATGCTCAAT

755

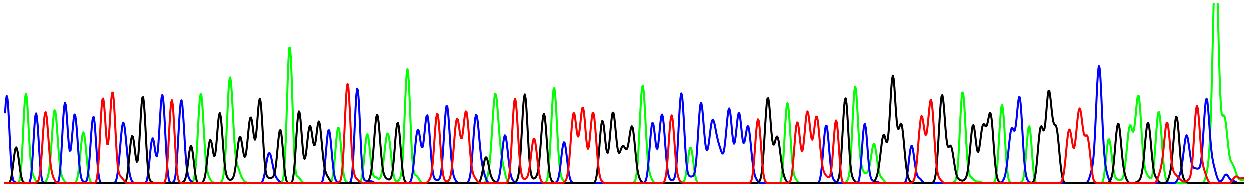

C CGT GTAGGTATGTGGTCCTTGACTCTGCCACCCTGGCCTACGTGACGTGTCAGGACCTTCGTAGCATTGCAGACCCTGCAGAGCAGATGGTTATGGTGATCAAAGCCCCTCCTGAGACCCAGCTCCAAGCCGTGGACTCTTCGGAGGT
C CGT AAAGGTATGTGGTCCTTGACTCTGCCACCCTGGCCTACGTGACGTGTCAGGACCTTCGTAGCATTGCAGACCCTGCAGAGCAGATGGTTATGGTGATCAAAGCCCCTCCTGAGACCCAGCTCCAAGCCGTGGACTCTTCGGAGGT

1

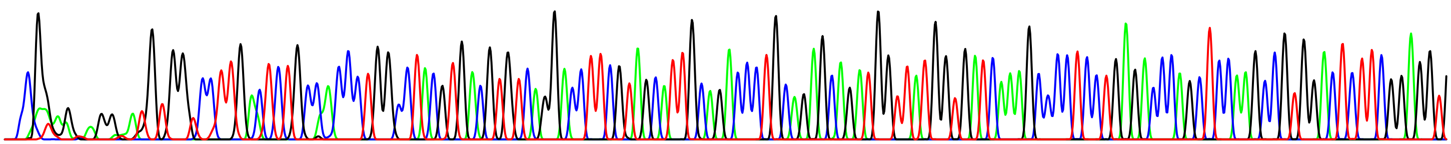

GAGATCTGGGAACTCCGGGCCCAACTGGGCTGGGCTGGGCTGGGCTGGTGGTCACCTGGGCCTCAGTTTACCCTGCCTGCTGCTTCCACCCAGAACTTTCAGATCTCCCTTAAGAGCAAACAAGGCCCGATCGATGTTTTCCTGTGCCCTGA
GAGATCTGGGAACTCCGGGCCCAACTGGGCTGGGCTGGGCTGGGCTGGTGGTCACCTGGGCCTCAGTTTACCCTGCCTGCTGCTTCCACCCAGAACTTTCAGATCTCCCTTAAGAGCAAACAAGGCCCGATCGATGTTTTCCTGTGCCCTGA

150

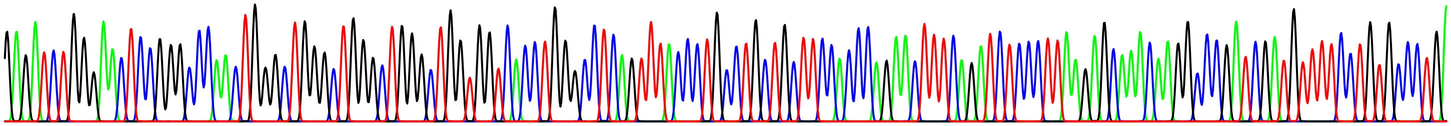

GGAGACCGTAGGTGGGATCAGCCCTGGGAAGACCCCATCCCAGGAGGTCACTTCTGAGGAGGAGAACAGGGCCACTGACTCTGCCACCATAGTGTCACCACCACCATCATCTCCCCCCTCATCCCTCACCACAGATCCCAGCCAGTCTCTAC
GGAGACCGTAGGTGGGATCAGCCCTGGGAAGACCCCATCCCAGGAGGTCACTTCTGAGGAGGAGAACAGGGCCACTGACTCTGCCACCATAGTGTCACCACCACCATCATCTCCCCCCTCATCCCTCACCACAGATCCCAGCCAGTCTCTAC

302

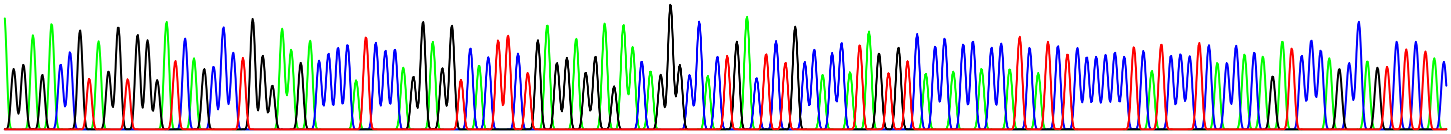

TCAGCCTGGAGCAAGGTGGGTGATGGGTAGGTGGGTGGGGTGGGGCAGGGCCCCTCTTCTGGGGGGTGGGCAGGCACGACAGCCCCTGCCTTCCTCCCTGCTGGGGCATCCCCGGCCTGTGATGCTCCCCGTCTCCCCAGAACCGCTGTT
TCAGCCTGGAGCAAGGTGGGTGATGGGTAGGTGGGTGGGGTGGGGCAGGGCCCCTCTTCTGGGGGGTGGGCAGGCACGACAGCCCCTGCCTTCCTCCCTGCTGGGGCATCCCCGGCCTGTGATGCTCCCCGTCTCCCCAGAACCGCTGTT

454

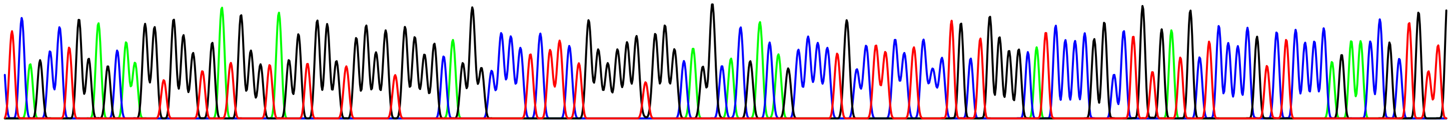

GTCCCGGATGGGCAGCCTGCGGGCTCCCGTGGACGAGGACCGCCTGTCCCCGCTGGTGGCGGCCGACTCGCTCCTGGAGCATGTGCGGGAGGACTTCTCCGGCCTCCTCCCTGAGGAGTTCATCAGCCTTTCCCCACCCCACGAGGCCCT
GTCCCGGATGGGCAGCCTGCGGGCTCCCGTGGACGAGGACCGCCTGTCCCCGCTGGTGGCGGCCGACTCGCTCCTGGAGCATGTGCGGGAGGACTTCTCCGGCCTCCTCCCTGAGGAGTTCATCAGCCTTTCCCCACCCCACGAGGCCCT

604

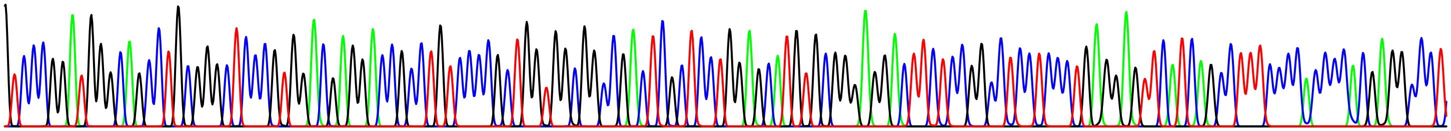

CGACTACCACTTCGGCCTCGAGGAGGGCGAGGGCATCAGAGACCTCTTCGACTGTGACTTTGGGGACCTCACCCCCCTGGATTTCTGACAGG CTTGGAGGGACCAGG TTCAGAAAAATGCTCAA C
CGACTACCACTTCGGCCTCGAGGAGGGCGAGGGCATCAGAGACCTCTTCGACTGTGACTTTGGGGACCTCACCCCCCTGGATTTCTGACAGG CTTGGAGGGACCAGG TTCAGAAAAATGCTCAA C

754

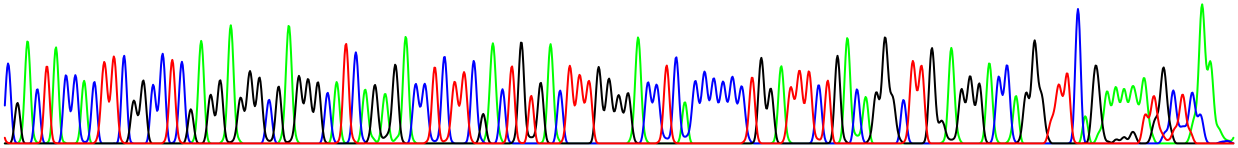

Supplement: Supplementary file 2 — E2F1 exons sequencing traces [file 41388_2022_2251_MOESM2_ESM.pdf]
